# Differential Effects of Losartan and Finerenone on Diabetic Remodeling, Oxidative Stress and ACE Activity in the Gastrointestinal Tract of Streptozotocin-Induced Diabetic Rats

**DOI:** 10.3390/ijms26136294

**Published:** 2025-06-29

**Authors:** Marisa Esteves-Monteiro, Cláudia Vitorino-Oliveira, Joana Castanheira-Moreira, Mariana Ferreira-Duarte, Patrícia Dias-Pereira, Vera Marisa Costa, Manuela Morato, Margarida Duarte-Araújo

**Affiliations:** 1 Associated Laboratory for Green Chemistry of the Network of Chemistry and Technology (LAQV@REQUIMTE), University of Porto, 4050-313 Porto, Portugal; mariana.mfd@gmail.com (M.F.-D.); pdiaspereira@yahoo.com.br (P.D.-P.); mmorato@ff.up.pt (M.M.); 2 Department of Immuno-Physiology and Pharmacology, School of Medicine and Biomedical Sciences, University of Porto (ICBAS-UP), 4050-313 Porto, Portugal; up201907029@edu.icbas.up.pt; 3 Laboratory of Pharmacology, Department of Drug Sciences, Faculty of Pharmacy, University of Porto (FFUP), 4050-313 Porto, Portugal; 4 Institute for Health and Bioeconomy (i4HB), Laboratory of Toxicology, Department of Biological Sciences, FFUP, 4050-313 Porto, Portugal; claudiaisabel1957@hotmail.com (C.V.-O.); veramcosta@ff.up.pt (V.M.C.); 5 Applied Molecular Biosciences Unit (UCIBIO), Laboratory of Toxicology, Department of Biological Sciences, FFUP, 4050-313 Porto, Portugal; 6 Department of Pathology and Molecular Immunology, ICBAS-UP, 4050-313 Porto, Portugal

**Keywords:** diabetes, gastrointestinal, STZ rats, angiotensin converting enzyme, glutathione, losartan, finerenone

## Abstract

Gastrointestinal (GI) complications are common in diabetes, but the role of the local renin-angiotensin-aldosterone system (RAAS) in gut remodeling remains unclear. This study examined histomorphometric alterations, oxidative stress, and systemic and tissue-specific angiotensin converting enzyme (ACE) and ACE2 activity in streptozotocin (STZ)-induced diabetic rats. Adult male Wistar rats (*n* = 24) were assigned to control (CTRL), diabetic (STZ), and diabetic groups treated with losartan (STZ-LOS, 20 mg/kg/day) or finerenone (STZ-FIN, 10 mg/kg/day). After 14 days, gut samples were collected from the stomach, duodenum, jejunum, ileum, and colon for histology, glutathione measurements (GSH/GSSG), and ACE/ACE2 activity assessment. Diabetic rats exhibited increased GI wall thickness—particularly in the mucosal and muscular layers—elevated GSSG levels, and a reduced GSH/GSSG ratio. Losartan prevented these changes, whereas finerenone did not produce a significant effect. Circulating ACE and ACE2 levels were elevated, but the ACE2/ACE ratio remained unchanged. Locally, ACE activity increased across gut segments, whereas ACE2 remained stable, lowering the ACE2/ACE ratio, particularly in the duodenum and jejunum. The Z-FHL/h-HL ratio was above 1 across segments but decreased in these same regions (jejunum and duodenum). These findings highlight the protective role of losartan against diabetic GI remodeling via AT_1_R blockade and suggest complex, segment-specific RAAS regulation in diabetic gut pathology.

## 1. Introduction

Diabetes is a serious chronic disorder and one of the fastest-growing global health emergencies of the 21st century [1]. There are two main forms of diabetes: type 1 (T1D) and type 2 (T2D). Although T2D is vastly more common, T1D is usually more serious, as it is caused by the destruction of pancreatic β-cells, leading to an absolute insulin deficiency [2,3]. Due to the high prevalence and impact of diabetes, and the essential role of animal models in research, using these models to study the disease’s mechanisms and test treatments is crucial [4]. The streptozotocin (STZ) model is widely used to induce T1D in rodents, as STZ selectively destroys pancreatic β-cells and produces structural, functional, and biochemical changes analogous to those observed in human diabetes [5].

Gastrointestinal (GI) complications of diabetes are highly prevalent and constitute a significant cause of morbidity, affecting up to 75% of diabetic patients, which influence their health status and quality of life [6,7]. However, awareness of these complications among physicians is often limited, with limited treatment options available [8,9]. Diabetic intestinal dysfunction appears multifactorial, involving advanced glycation end-product (AGE) accumulation, enteric nervous system damage, and smooth muscle fibrosis contributing to GI tract remodeling with reduced elasticity and impaired intestinal wall compliance [10]. These GI complications often lead to conditions like gastroparesis, enteropathy, and colonic disorders (e.g., chronic constipation and diarrhea) [11]. Despite these alterations being described since 1971 [12], the precise mechanism underlying diabetes-related GI complications remains far less understood compared to diabetic retinopathy or nephropathy [13]. Consequently, many patients remain undiagnosed and untreated [14].

The renin–angiotensin-aldosterone system (RAAS) is best known for its role in cardiovascular and renal health, but it is also active in the GI tract, where its effector peptide, angiotensin II (Ang II), acts primarily via the Ang II type 1 receptor (AT_1_R) to influence smooth muscle contraction in the intestine and colon [15,16]. Ang II is primarily formed from Ang I by angiotensin converting enzyme (ACE), and promotes vasoconstriction, inflammation, and fibrosis [17]. Conversely, ACE2 metabolizes Ang II into Ang 1–7, which counteracts these effects. Additionally, ACE2 converts Ang I into Ang 1–9, later processed by ACE into Ang 1–7 [18]. ACE2 has a single catalytic domain, whereas ACE has two (N- and C-domains) with distinct specificities: the C-domain primarily hydrolyzes Ang I into Ang II, while the N-domain metabolizes Ang 1–7 into Ang 1–5 and other peptides [19,20]. The balance between ACE and ACE2 activities reflects RAAS regulation, orchestrating the interplay between its classic and counter-regulatory pathways [21]. Moreover, the RAAS is no longer seen solely as a circulating hormonal system; it is now recognized for its local action as well [22]. Various tissues, including the GI tract, can synthesize key components of the RAAS, containing all components necessary for the production of Ang II [23]. We have previously described a decreased response to Ang II in the ileum and colon of STZ-induced diabetic rats [24]. Moreover, there is a close relationship between diabetes and RAAS, with RAAS contributing significantly to the development of several diabetic complications [25]. It has been shown, in diabetic patients, an overactivation of the RAAS with increased levels of circulating and tissue ACE, especially in the cardiovascular and renal systems, leading to the increased production of Ang II and AT_1_R activation, contributing to increased oxidative stress and fibrosis in several organs [26,27]. But, to date, no one considered the possible therapeutic role of RAAS on diabetic impairment of intestinal motility.

Various pathways contribute to tissue damage via RAAS, and one includes heightened oxidative stress, characterized by elevated levels of reactive oxygen species (ROS) [28]. Moreover, chronic hyperglycemia is linked to decreased cellular levels of glutathione (GSH) [29]. GSH is the most powerful antioxidant in the organism; it undergoes oxidation to GSSG (glutathione disulfide). GSSG is subsequently regenerated back to GSH by GSH-reductase, using NADPH as a cofactor. This dynamic interconversion between GSH and GSSG is crucial for regulating redox-dependent cell signaling [30]. The excessive production of ROS prompts GSSG increase, thus altering the ratio between GSH and GGSG [31]. Maintaining an optimal ratio of GSH to GSSG within the cell is crucial for survival, and a decrease in this ratio may be used as a marker of oxidative stress [32]. Oxidative stress and ROS formation has already been described as being markedly increased by uncontrolled hyperglycemia [33], and oxidative stress has already been identified as a significant contributor to gastrointestinal dysmotility, including post-operative ileus and diabetic gastroparesis [34]. Also, our group recently showed a lower total GSH (tGSH) and GHS/GSSG ratio in the gut of a long-term model of T2D [35].

Given the limited understanding of local RAAS in gut remodeling—particularly under diabetic conditions—this study aimed to evaluate the preventive effects of losartan an AT_1_ receptor blocker (ARB), and finerenone, a selective mineralocorticoid receptor antagonist (MRA), on GI remodeling and oxidative stress in STZ-induced diabetic rats. To characterize the circulating and local RAAS, the ACE/ACE2 activity balance in the serum and GI tract was also assessed.

## 2. Results

### 2.1. Animal Monitorization

**Figure 1:**
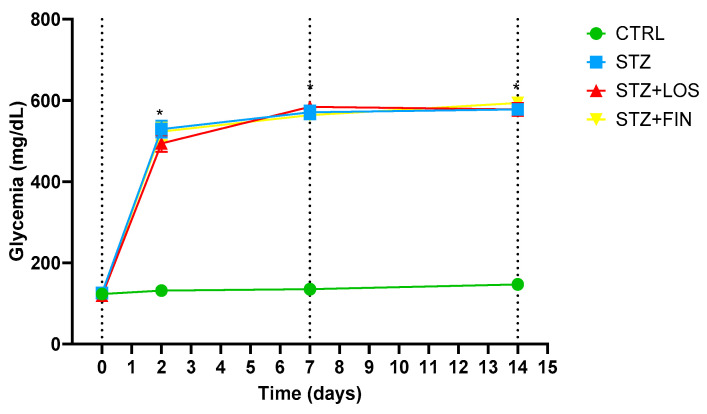
Blood glucose concentrations of control (CTRL, *n* = 8), non-treated streptozotocin-induced (STZ, *n* = 8), streptozotocin-induced treated with losartan (STZ+LOS, *n* = 8) and streptozotocin-induced treated with finerenone (STZ+FIN, *n* = 8) rats measured on day 0, day 2, day 7, and day 14. Values are presented as mean ± SD. * Statistical difference to CTRL, *p* < 0.05.

Before induction, the basal glycemia of the control and STZ-induced rats, treated and non-treated, was similar (CTRL: 122.75 ± 4.28 mg/dL vs. STZ: 125.5 ± 5.10 mg/dL vs. STZ+LOS: 119.13 ± 3.42 vs. STZ+FIN: 122.88 ± 6.50, *n* = 32 divided equally across the four experimental groups (*n* = 8 *per* group), *p* > 0.05) (Figure 1). Induced rats had their initial glycemia increased to 529.5 ± 20.76 mg/dL (STZ), 494.25 ± 20.73 mg/dL (STZ+LOS) and 523.38 ± 21.46 mg/dL (STZ+FIN) within 48 h (*p* < 0.0001), while in the control rats glycaemia was roughly the same within 48 h (131.63 ± 4.93 mg/dL; *p* > 0.05). At day 7 and day 14, almost all STZ rats presented glycemia above 600 mg/dL with ketone bodies, while control animals presented glycemic values of 142.5 ± 4.96 mg/dL on the 14th day. The glucometer used was selected because it could quantify blood glucose values up to 600 mg/dL, with values above this threshold being considered “HIGH”. In this study, for graphic purposes we considered all “HIGH” values to correspond to 600 mg/dL, a very high value which raises no doubt about the diabetic condition the animals were in throughout the protocol.

**Figure 2:**
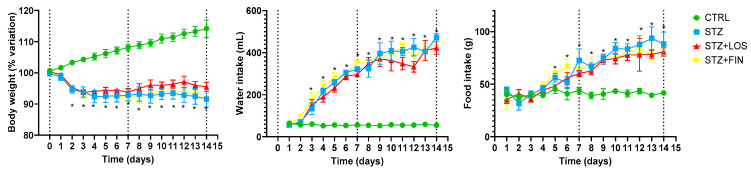
Evaluation during the experimental protocol (14 days) in control (CTRL, *n* = 8), streptozotocin-induced diabetic rats (STZ, *n* = 8), STZ diabetic rats treated with losartan (STZ+LOS, *n* = 8) and STZ diabetic rats treated with finerenone (STZ+FIN, *n* = 8) of: body weight, % of body weight variation, food intake, and water intake. Values are mean ± SD. * Statistical difference to CTRL, *p* < 0.05.

The parameters documented during the daily monitorization (% of body weight loss, water intake, food intake) are shown in Figure 2.

In the control group, rats progressively gained weight, their weight being 14.21 ± 2.78% higher by d14 than on d0 (before fasting). All of the diabetic rats had a consistent weight loss that was more pronounced on d2 (less 5% compared to the initial weight) and then maintained that relatively stable lower weight. The loss of body weight was not affected by any of the treatments applied. The % of body weight variation was 8.45 ± 2.53% (STZ), 4.52 ± 1.51% (STZ+LOS) and 8.2 ± 1.5% (STZ+FIN) lower at d14 when compared to the initial weight (before fasting) (Figure 2).

The food intake was significantly higher in all three groups of diabetic rats than the controls after d4. The STZ rats started the experimental protocol eating 44.67 ± 2.63 g on the first day, and progressively increased food consumption until the last day, when their intake was 88.33 ± 11.41 g/cage/day. STZ+LOS rats started the experimental protocol eating 35.5 ± 3.71 g on the first day, and progressively increased food consumption until the last day, when their intake was 80.85 ± 3.94 g/cage/day. STZ+FIN rats started the experimental protocol eating 33.67 ± 7.26 g on the first day, and progressively increased food consumption until the last day, when the intake was 80.25 ± 5.15 g/cage/day. The control group maintained a constant food intake during the experimental time, with a mean consumption of 41.22 ± 3.38 g/cage/day. The four groups were always fed at libitum.

As expected, water intake was significantly higher in all diabetic groups than in the controls, which maintained a relatively constant water intake throughout the experimental protocol: 56.44 ± 3.53 mL/cage/day. All diabetic rats exhibited increased water consumption starting on day 3, with intake progressively rising throughout the protocol, eventually reaching levels more than seven times higher than those of the control group (at day 14: STZ: 472.75 ± 19.69 mL/cage/day; STZ+LOS: 423 ± 30.08 mL/cage/day; STZ+FIN: 415 ± 22.29 mL/cage/day).

### 2.2. Macroscopic Evaluation

**Figure 3:**
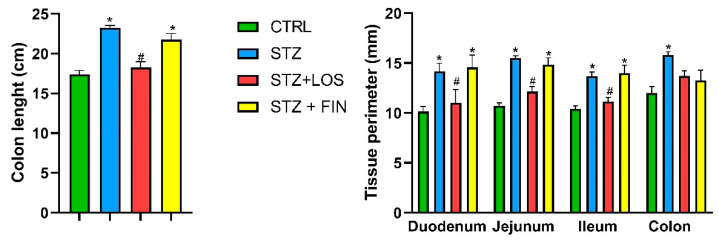
Macroscopic evaluation: colon length (cm) and tissue perimeter (mm) of duodenum, jejunum, ileum and colon of control (CTRL, *n* = 8), streptozotocin-induced diabetic rats (STZ, *n* = 8), streptozotocin-induced diabetic rats treated with losartan (STZ+LOS, *n* = 8) and streptozotocin-induced diabetic rats treated with finerenone (STZ+FIN, *n* = 8). Values are mean ± SD. * Statistical difference (*p* < 0.05) to CTRL; ^#^ Statistical difference (*p* < 0.05) to STZ.

Colon length was significantly greater in the STZ group (23.35 ± 0.31 cm) compared to the CTRL group (17.38 ± 0.50 cm). Losartan prevented this increase, as the colon length in the STZ+LOS group (18.25 ± 0.73 cm) was comparable to the CTRL group. In contrast, finerenone treatment did not mitigate the increase, as colon length in this group (21.75 ± 0.75 cm) remained significantly different from the control group but was comparable to the STZ group (Figure 3).

We also measured the circumferential perimeter of all intestinal segments and found it to be significantly greater in the STZ group across all segments compared to the control group (in mm: duodenum: 14.17 ± 0.83 vs. 10.14 ± 0.51; jejunum: 15.5 ± 0.22 vs. 10.71 ± 0.29; ileum: 13.67 ± 0.42 vs. 10.43 ± 0.30; colon: 15.83 ± 0.31 vs. 12.00 ± 0.65; *p* < 0.05 for all). Interestingly, treatment with losartan effectively prevented this increase in all segments (duodenum: 11 ± 0.36; jejunum: 12.14 ± 0.51; ileum: 11.14 ± 0.40; colon: 13.7 ± 0.52; *p* < 0.05 for all compared to CTRL). In contrast, finerenone treatment did not alter this increase (duodenum: 14.57 ± 1.13; jejunum: 14.85 ± 0.87; ileum: 14 ± 0.79; *p* < 0.05 for all compared to CTRL), except for the colon (13.25 ± 1.03, *p* > 0.05) (Figure 3).

### 2.3. Microscopic Evaluation

**Figure 4:**
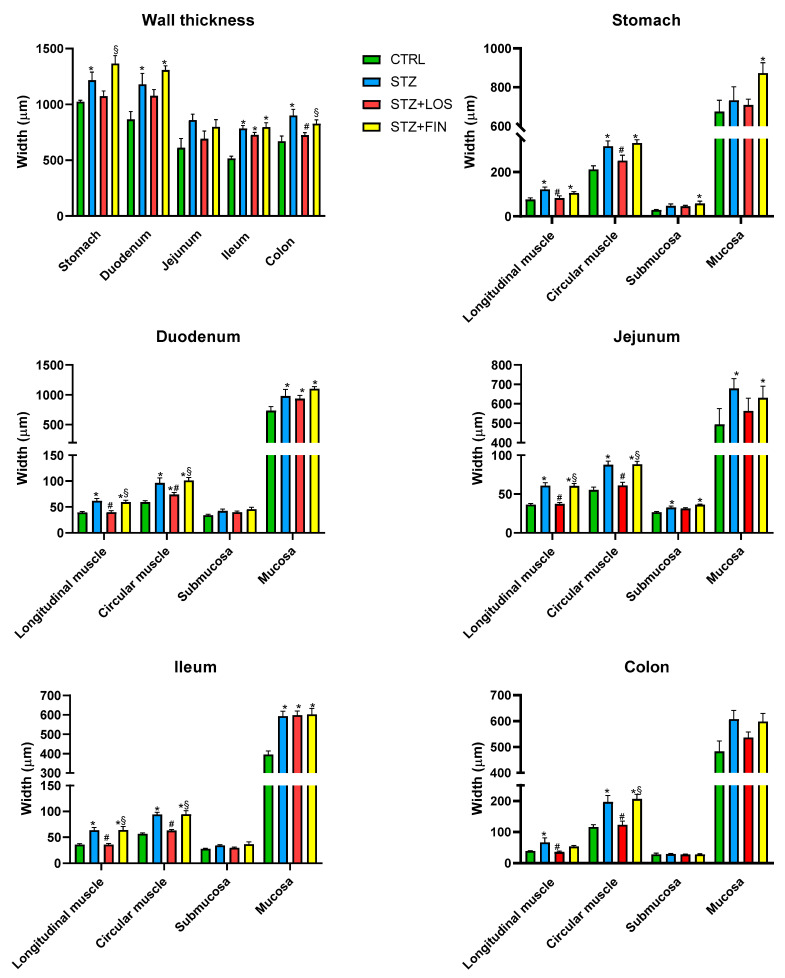
Microscopic evaluation of the gastrointestinal walls of the stomach, duodenum, jejunum, ileum, and colon and thickness (μm) of the intestinal layers (longitudinal muscle, circular muscle, submucosa and mucosa) of each intestinal segment of control (CTRL, *n* = 8), streptozotocin-induced diabetic rats (STZ, *n* = 8), streptozotocin-induced diabetic rats treated with losartan (STZ+LOS, *n* = 8) and streptozotocin-induced diabetic rats treated with finerenone (STZ+FIN, *n* = 8). Values are mean ± SD. * Statistical difference (*p* < 0.05) to CTRL; ^#^ Statistical difference (*p* < 0.05) to STZ; ^§^ Statistical difference (*p* < 0.05) to STZ+LOS.

The results of the histological evaluation of the intestinal segments from all groups (Figure 4) were consistent with the macroscopic data previously described, revealing an increase in the thickness of the intestinal layers in the duodenum, jejunum, ileum, and colon of STZ animals compared to controls, and its prevention by losartan but not by finerenone.

Considering the wall thickness as a whole, STZ segments showed an increase in the thickness compared to controls in the stomach (1216.28 ± 73.64 µm vs. 1023.89 ± 13.38 µm), duodenum (1178.89 ± 99.59 µm vs. 866.23 ± 69.34 µm), ileum (784.39 ± 26.65 µm vs. 515.19 ± 20.01 µm), and colon (900.13 ± 55.43 µm vs. 669.78 ± 47.06 µm), but not in the jejunum (859.42 ± 53.80 vs. 611.83 ± 83.16, *p* > 0.05). Treatment with losartan was only able to prevent this increase in the colon (724.27 ± 22.61 µm). Finerenone treatment did not have any effect on the walls’ thickness.

Both muscular layers (longitudinal muscle—LM and circular muscle—CM) were increased in all the segments studied in STZ animals compared to controls (LM, in µm: stomach: 121.30 ± 10.28 vs. 75.64 ± 7.57; duodenum: 61.78 ± 4.57 vs. 39.44 ± 2.1; jejunum: 60.89 ± 3.92 vs. 36.37 ± 1.37; ileum: 63.60 ± 5.18 vs. 35.72 ± 1.69; colon: 66.29 ± 14.91 vs. 38.75 ± 1.07; CM, in µm: stomach: 315.56 ± 23.84 vs. 211.19 ± 16.13; duodenum: 96.44 ± 9.8 vs. 59.24 ± 2.77; jejunum: 87.67 ± 4.9 vs. 55.29 ± 3.69; ileum: 94.08 ± 4.25 vs. 56.59 ± 1.81; colon: 196.64 ± 20.72 vs. 115.69 ± 7.84, respectively, *p* < 0.05 for all). Treatment with losartan successfully prevented this thickness increase in all the portions studied (LM, in µm: stomach: 82.21 ± 9.94; duodenum: 39.95 ± 2.87; jejunum: 37.27 ± 1.97; ileum: 35.85 ± 2.22; colon: 35.92 ± 2.66; CM, in µm: stomach: 251.15 ± 24.28; jejunum: 60.97 ± 4.21; ileum: 63.03 ± 2.00; colon: 123.06 ± 11.84, *p* > 0.05 for all compared to CTRL) except in the CM of the duodenum where the STZ-induced increase was only attenuated but still not similar to CTRL (73.88 ± 4.06, *p* < 0.05 compared to CTRL and STZ). Finerenone’s treatment did not prevent the reported increase and the results did not differ from those in the CTRL group (LM, in µm: stomach: 105.01 ± 6.14; duodenum: 59.28 ± 3.5; jejunum: 60.4 ± 3.31; ileum: 63.97 ± 6.67; colon: 53.49 ± 3.16; CM, in µm: stomach: 330.07 ± 14.94; duodenum: 101.19 ± 5.8; jejunum: 88.35 ± 3.95; ileum: 94.47 ± 6.9; colon: 206.19 ± 15.18, *p* < 0.05 for all compared to CTRL).

The mucosa was increased in STZ animals compared to controls in duodenum (978.47 ± 111.55 µm vs. 733.59 ± 67.50 µm), jejunum (678.36 ± 51.51 µm vs. 493.57 ± 81.18 µm) and ileum (592.65 ± 26.59 µm vs. 395.19 ± 18.02 µm), but none of the treatments employed was able to prevent this alteration.

In general, submucosa presented no differences between all groups, with exception of STZ and STZ+FIN in jejunum where there was an increase in the thickness compared to CTRL group (*p* < 0.05).

**Figure 5:**
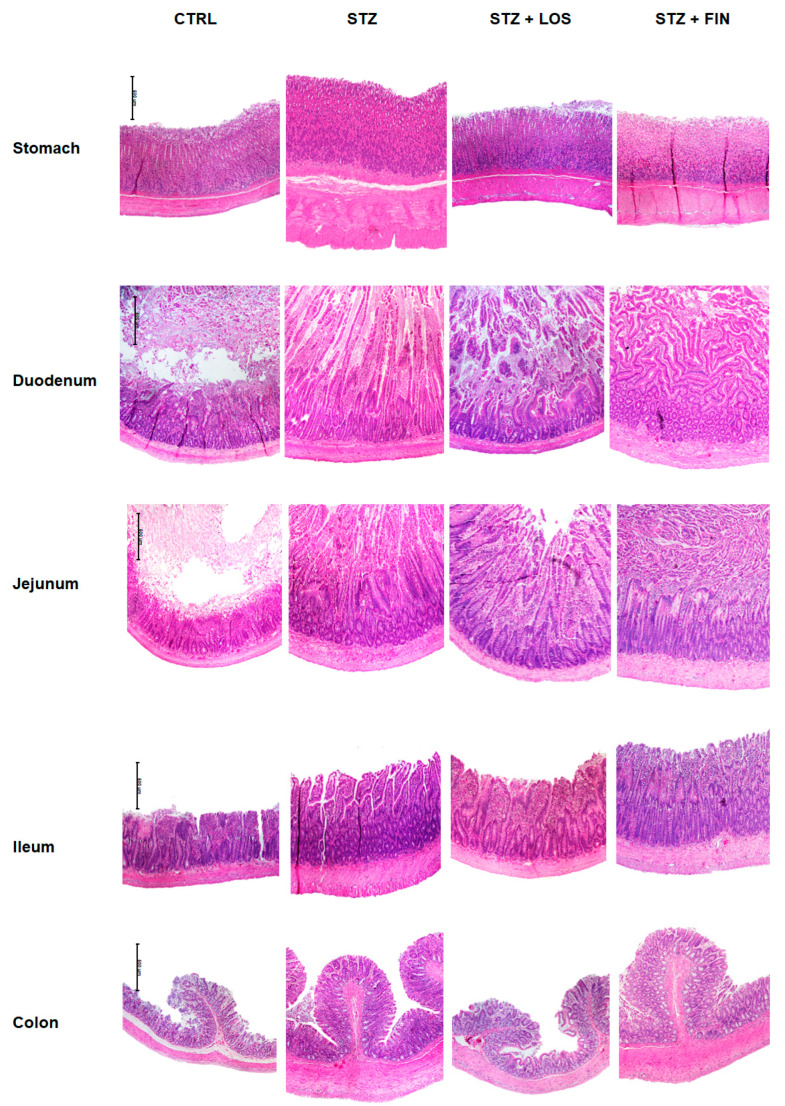
Representative microscopic photographs of stomach, duodenum, jejunum, ileum, and colon of control (CTRL), streptozotocin (STZ)- induced (STZ), STZ treated with losartan (STZ+LOS), and STZ treated with finerenone (STZ+FIN) stained with hematoxylin and eosin, captured using a 40× magnification. The scale bar (500 µm) shown in the first image of each row applies to all images in that row; *n* = 8 *per* group.

Representative images of CTRL, STZ, STZ+LOS, and STZ+FIN animals are shown in Figure 5, covering all the sections studied. These images offer a detailed visual comparison of the differences in each analyzed area.

### 2.4. Total GSH and GSSG Quantification

**Figure 6:**
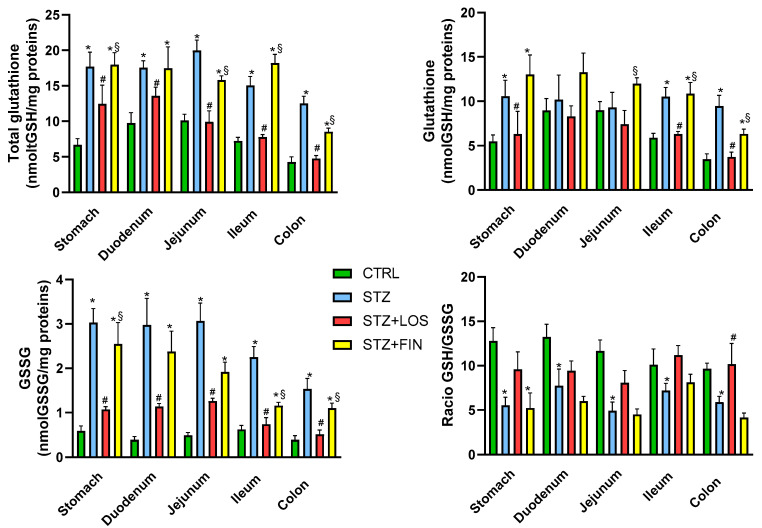
Glutathione evaluation of gastrointestinal segments (stomach, duodenum, jejunum, ileum and colon) of control rats (CTRL, *n* = 8), streptozotocin-induced diabetic rats (STZ, *n* = 8), streptozotocin-induced diabetic rats treated with losartan (STZ+LOS, *n* = 8) and streptozotocin-induced diabetic rats treated with finerenone (STZ+FIN, *n* = 8): total glutathione (tGSH) quantification (nmol tGSH/mg protein); glutathione (nmol GSH/mg protein), oxidized glutathione (GSSG) quantification (nmol GSSG/mg protein) and ratio GSH/GSSG. Values are mean ± SD. * Statistical difference (*p* < 0.05) to CTRL; ^#^ Statistical difference (*p* < 0.05) to STZ; ^§^ Statistical difference (*p* < 0.05) to STZ+LOS.

To investigate the potential causes of the remodeling observed earlier, we measured tGSH and GSSG levels as indicators of redox index and putative oxidative stress, a key factor in the development of diabetic complications. The results of the tGSH quantification revealed an increase in all regions of the diabetic animals compared to the controls (in nmol tGSH/mg protein: stomach: 17.69 ± 2.05 vs. 6.67 ± 0.88; duodenum: 17.56 ± 0.96 vs. 9.73 ± 1.48; jejunum: 19.96 ± 1.49 vs. 10.11 ± 0.88; ileum: 15.03 ± 1.28 *vs*. 7.23 ± 0.5; colon: 12.52 ± 1.01 vs. 4.25 ± 0.74, respectively, *p* < 0.05 for all). Treatment with losartan prevented this increase in all regions studied (in nmol tGSH/mg protein, duodenum: 13.57 ± 1.24; jejunum: 9.93 ± 1.53; ileum: 7.79 ± 0.35; colon: 4.75 ± 0.34, *p* > 0.05 compared to CTRL), except in the stomach, where tGSH was lower than in the STZ group but still higher than in the CTRL group (12.45 ± 2.64 nmol/mg protein, *p* < 0.05 compared to both CTRL and STZ). In contrast, treatment with finerenone did not affect these alterations in any of the regions studied (in nmol tGSH/mg protein: stomach: 17.97 ± 1.71; duodenum: 17.47 ± 3.02; jejunum: 15.8 ± 0.61; ileum: 18.19 ± 1.24, *p* < 0.05 compared to CTRL) except in the colon, where tGSH levels were decreased compared to STZ but increased compared to CTRL (8.53 ± 0.5 nmol/mg protein, *p* < 0.05 compared to both CTRL and STZ) (Figure 6). GSH levels were also elevated in diabetic animals compared to the controls, but only in the stomach (10.58 ± 1.79 nmol/mg protein vs. 5.48 ± 0.73 nmol/mg protein), ileum (10.53 ± 1.03 nmol/mg protein vs. 5.89 ± 0.49 nmol/mg protein), and colon (9.46 ± 1.21 nmol/mg protein vs. 3.47 ± 0.60 nmol/mg protein). Treatment with losartan prevented this increase in all affected regions (stomach: 6.31 ± 2.57 nmol/mg protein; ileum: 6.31 ± 0.30 nmol/mg protein; colon: 3.73 ± 0.54 nmol/mg protein, *p* > 0.05 compared to both CTRL), while treatment with finerenone had no effect.

Similar to what was observed with tGSH, GSSG levels were also elevated in all regions of the STZ animals compared to the controls (in nmol GSSG/mg protein, stomach: 3.03 ± 0.32 vs. 0.59 ± 0.11; duodenum: 2.98 ± 0.60 vs. 0.39 ± 0.07; jejunum: 3.07 ± 0.40 vs. 0.49 ± 0.06; ileum: 2.25 ± 0.24 vs. 0.62 ± 0.09; colon: 1.53 ± 0.24 vs. 0.39 ± 0.09, respectively, *p* < 0.05 for all). Treatment with losartan prevented this increase in all of the regions studied (in nmol GSSG/mg protein, stomach: 1.07 ± 0.07; duodenum: 1.14 ± 0.07; jejunum: 1.26 ± 0.06; ileum: 0.74 ± 0.15; colon: 0.51 ± 0.10, *p* > 0.05 compared to CTRL). In contrast, treatment with finerenone did not affect these alterations in the stomach (2.55 ± 0.48 nmol/mg protein), duodenum (2.38 ± 0.46 nmol/mg protein), and colon (1.10 ± 0.12 nmol/mg protein), but was able to attenuate GSSG increase values in the jejunum (1.92 ± 0.22 nmol/mg protein) and ileum (1.16 ± 0.07 nmol/mg protein) (*p* < 0.05 compared to both CTRL and STZ) (Figure 6).

Regarding the GSH/GSSG ratio, a decrease was observed in all portions of the STZ diabetic rats compared to the controls (stomach: 5.53 ± 0.90 vs. 12.77 ± 1.52; duodenum: 7.73 ± 1.9 vs. 13.22 ± 1.45; jejunum: 4.93 ± 0.97 *vs*. 11.65 ± 1.27; ileum: 7.19 ± 0.82 vs. 10.1 ± 1.79; colon: 5.89 ± 0.65 vs. 9.64 ± 0.66, respectively, *p* < 0.05 for all). However, in the losartan-treated animals, the ratio was similar to the controls in all portions studied (stomach: 9.58 ± 1.98; duodenum: 9.42 ± 1.11; jejunum: 8.08 ± 1.37; ileum: 11.19 ± 1.09; colon: 10.15 ± 2.36, *p* > 0.05). Finerenone-treated animals presented a lower ratio compared to the controls in all portions (stomach: 5.22 ± 1.71; duodenum: 6.00 ± 0.55; jejunum: 4.50 ± 0.63; colon: 4.16 ± 0.49, *p* < 0.05 for all), except the ileum (8.11 ± 0.94, *p* > 0.05) (Figure 6).

### 2.5. ACE and ACE 2 Activity

**Figure 7:**
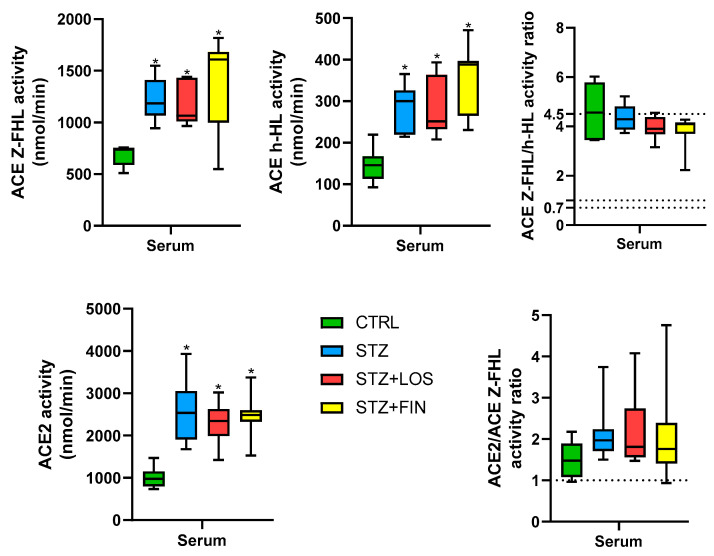
Angiotensin Converting Enzyme (ACE) catalytic activities, including Z-FHL and h-HL substrates, as well as the ACE Z-FHL/h-HL ratio and the ACE2/ACE activity ratio in the serum of control rats (CTRL, *n* = 8), streptozotocin-induced diabetic rats (STZ, *n* = 8), and streptozotocin-induced diabetic rats treated with losartan (STZ+LOS, *n* = 8) or finerenone (STZ+FIN, *n* = 8). Values are median [95% confidence limits]. * Statistical difference (*p* < 0.05).

Both ACE Z-FHL and ACE h-HL activities were higher in the serum of all diabetic groups compared to the controls (ACE Z-FHL activity in nmol/min, CTRL: 737.7 [605.5, 771.3]; STZ: 1186 [1058, 1398]; STZ+LOS: 1056 [984.1, 1333]; STZ+FIN: 1609 [1007, 1752]; ACE h-HL activity in nmol/min, CTRL: 146 [92.65, 219.5]; STZ: 300.4 [214.4, 365.6]; STZ+LOS: 251.6 [208.1, 393.7]; STZ+FIN: 388.1 [230.7, 471.1], *p* < 0.05 for all). Also, the ACE Z-FHL/h-HL activity ratio was greater than 1 and consistently close to 4, indicating a predominant N-domain activity in all of the experimental groups (Figure 7).

ACE2 activity was higher in the serum of all diabetic groups compared to controls (ACE2 activity in nmol/min, CTRL: 976.4 [733.9, 1469]; STZ: 2537 [1679, 3932]; STZ+LOS: 2345 [1422, 3019]; STZ+FIN: 2486 [1527, 3374], *p* < 0.05 for all). Also, the ACE2/ ACE Z-FHL activity ratio was greater than 1 in all experimental groups (Figure 7).

**Figure 8:**
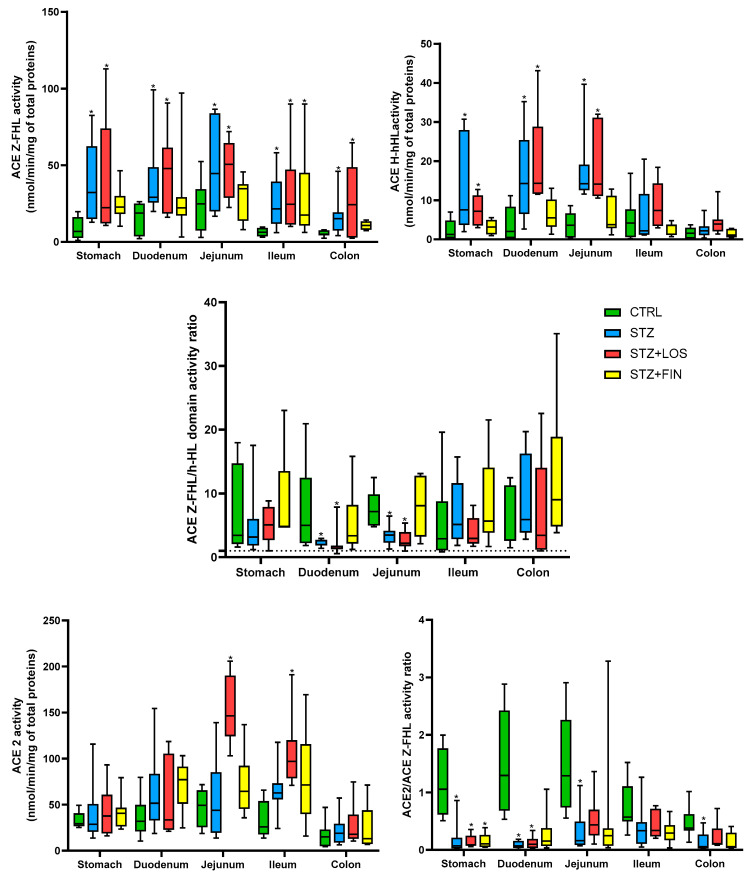
Angiotensin Converting Enzyme (ACE) catalytic activities, including Z-FHL and h-HL substrates, as well as the ACE Z-FHL/h-HL ratio and the ACE2/ACE activity ratio in the gastrointestinal tract (stomach, duodenum, jejunum, ileum and colon) of control rats (CTRL, *n* = 8), streptozotocin-induced diabetic rats (STZ, *n* = 8), and streptozotocin-induced diabetic rats treated with losartan (STZ+LOS, *n* = 8) or finerenone (STZ+FIN, *n* = 8). Values are median [95% confidence limits]. * Statistical difference (*p* < 0.05).

ACE Z-FHL activity was higher in the intestinal portions of STZ diabetic animals compared to controls across all studied sections (in nmol/min/mg of total proteins, stomach: 32.22 [12.85, 82.47] vs. 6.77 [0.95, 19.79]; duodenum: 29.9 [25.01, 99.15] vs. 18.76 [2.14, 26.21]; jejunum: 46.49 [23.42, 86.52] vs. 24.91 [3.03, 35.45]; ileum: 21.51 [11.18, 58.15] vs. 6.31 [3.07, 9.67]; colon: 15.13 [6.98, 45.97] vs. 5.07 [2.46, 7.93], respectively, *p* < 0.05 for all). Treatment with losartan did not affect this increase, as the intestinal portions of these animals exhibited ACE Z-FHL activity similar to that of the STZ animals and statistically different from the controls (in nmol/min/mg of total proteins, stomach: 25.26 [11.79, 112.8]; duodenum: 47.82 [18.02, 90.58]; jejunum: 50.54 [30.37, 68.61]; ileum: 24.52 [10.04, 90.00]; colon: 24.32 [2.53, 64.59], *p* < 0.05 for all). In the finerenone-treated animals, the ACE-Z-FHL activity in the ileum was higher than in the controls (17.57 [6.25, 89.9] nmol/min/mg of total proteins) differed from the controls (*p* < 0.05), while the remaining portions showed no significant difference compared to any of the other experimental groups (Figure 8).

Looking at ACE h-HL activity, it was higher in the intestinal portions of the STZ and LOS-treated animals compared to the controls in the stomach (in nmol/min/mg of total proteins, STZ: 7.53 [3.58, 30.71] and STZ+LOS: 7.19 [2.97, 12.75] vs. CTRL: 0.86 [0.15, 4.8], *p* < 0.05), duodenum in nmol/min/mg of total proteins, STZ: 14.27 [2.60, 35.21] and STZ+LOS: 20.71 [11.52, 44.51] vs. CTRL: 2.03 [0.11, 11.15], *p* < 0.05), and jejunum (in nmol/min/mg of total proteins, STZ: 13.87 [11.55, 39.64] and STZ+LOS: 19.47 [10.56, 31.78] vs. CTRL: 3.63 [0.24, 8.59], *p* < 0.05).

The ACE Z-FHE/h-HL activity ratio was greater than 1 in all of the experimental groups and higher than 4.5 in most of the portions, indicating an N-domain predominant activity. The results were similar between the four experimental groups studied, except for the duodenum and jejunum of the STZ and LOS-treated animals, which showed a decreased ratio compared to the controls (duodenum, STZ: 2.11 [1.39, 7.63] and STZ+LOS: 1.39 [0.56, 7.87] vs. CTRL: 9.37 [1.84; 24.83]; jejunum, STZ: 3.48 [1.29, 6.45] and STZ+LOS: 2.19 [0.96, 5.35] vs. CTRL: 8.03 [4.79; 13.80], *p* < 0.05) (Figure 8).

Contrary to what was observed in the serum, ACE2 activity was similar across all experimental groups, including diabetic animals, except in the jejunum and ileum of the losartan-treated diabetic animals. In these sections, ACE2 activity was higher than in the controls (in nmol/min/mg of total proteins, jejunum: 146.3 [103.1, 205.7] vs. 114.1 [27.94, 163.3]; ileum: 96.98 [70.92, 191.00] vs. 25.25 [17.50, 65.70], respectively, *p* < 0.05) (Figure 8).

The ACE 2/ ACE activity ratio was decreased in all diabetic portions compared to the controls (stomach: 0.07 [0.02, 0.86] vs. 1.06 [0.51, 5.00]; duodenum: 0.07 [0.01, 0.19] vs. 1.30 [0.54, 3.88]; jejunum: 0.16 [0.07, 1.19] vs. 1.30 [0.55, 2.91]; colon: 0.06 [0.02, 0.47] vs. 0.34 [0.13, 1.82], *p* < 0.05 for all), except ileum (*p* > 0.05). Losartan-treated animals also exhibited this decrease in the stomach (0.09 [0.06, 0.36]) and duodenum (0.1 [0.02, 0.32]), while only the stomach of finerenone-treated animals (0.11 [0.04, 0.39]) showed the same pattern (*p* < 0.05 for all) (Figure 8).

## 3. Discussion

This study is the first to demonstrate that losartan, an Ang II type 1 receptor blocker, effectively prevents both muscular hypertrophy and redox imbalance in the gastrointestinal tract of STZ-induced diabetic rats, providing compelling evidence for the central role of local RAAS activation—particularly Ang II signaling—in the structural and oxidative changes associated with diabetes. Diabetes is extensively known to induce significant alterations in the GI tract, as was already described by our and other groups [10,24,35]. This may contribute to the complications associated with the disease and decrease the quality of life of diabetic patients [10]. Our study provides novel insights through histopathological analysis, redox status evaluation, and the assessment of both systemic and local RAAS. Notably, the prevention of muscular hypertrophy and oxidative stress by losartan observed in this study highlights the critical role of AT_1_ receptor-mediated mechanisms in these pathological changes, offering new avenues for therapeutic intervention.

Before addressing the local effects of the treatments, it is important to revisit the systemic features of the diabetic model used in this study. As expected, the STZ-induced diabetic rats showed typical diabetes signs, including hyperglycemia, polyphagia, polydipsia, and weight loss [36]. These symptoms arise primarily due to a deficiency in insulin production due to selective destruction of pancreatic β-cells [37], leading to elevated blood glucose levels that disrupt normal metabolic functions [5]. The observed hyperglycemia triggers compensatory mechanisms such as polydipsia [38], polyphagia [39] and weight loss that was more pronounced on the second day, as already described by our [24] and other studies [40]. In this context, losartan and finerenone have been explored for their potential in managing diabetes-related complications [41,42], but their effects on the core diabetic symptoms remain under investigation as these medications do not appear to directly influence the classic symptoms of diabetes. Losartan may offer mild improvements in insulin sensitivity, but its action does not significantly lower blood glucose levels nor alleviate the compensatory mechanisms driven by hyperglycemia [43]. Similarly, finerenone’s role in managing renal complications in diabetes does not extend to improving glucose metabolism or reducing the symptoms associated with metabolic dysregulation [44]. Therefore, it is not surprising that no significant differences were observed in the typical diabetic signs between the STZ group and the two treated groups.

Among the systemic manifestations, polyphagia warrants particular attention because of its potential impact on GI morphology, including the increased colon length and intestinal perimeter previously reported in diabetic conditions [24,45]. Intestinal smooth muscle cells (SMC) demonstrate notable plasticity, adapting to functional demands through structural remodeling [45]. Jervis and colleagues [46] proposed that the enlargement of the intestine in diabetic animals may therefore be an adaptive response to polyphagia. In a study with diabetic rats induced by alloxan, these authors also described an enlargement of the diameter and length of the small intestine and colon [46]. Curiously, other causes of polyphagia like lactation [47] or hypothalamic lesions [48] may also induce GI enlargement—similar to the one seen in diabetes, reinforcing this theory. However, this hypothesis was challenged by another study, which showed that when the food intake of diabetic rats was matched to that of the controls, the results differed and (avoiding polyphagia) the intestinal mass of diabetic animals remained elevated [49]. Therefore, it seems prudent to assume that only part of the intestinal growth depends on polyphagia.

This macroscopic enlargement of the GI tract is further supported by histopathological evidence, which helps to distinguish mucosal and muscular contributions to the observed changes. Mucosal growth in diabetic animals may be partially driven by polyphagia, mediated by glucagon-like peptide 2 (GLP-2), which stimulates epithelial proliferation and contributes to intestinal growth [50]. The mucosal thickening also appears to be involved with a suppression of apoptosis that happens in the first week after STZ injection, returning to normal 3 weeks after, suggesting a transient effect [51]. Accumulation of AGE and RAGE in the mucosa may also contribute to increased mucosa thickness [52], which can further impair digestive function by altering the properties of the intestinal epithelial cells and the activity of digestive enzyme [53].

In parallel with mucosal adaptations, the muscular layer of the GI wall undergoes distinct and extensive remodeling in diabetes, associated with an increased production and accumulation of collagen type I and AGE/RAGE [10] and SMCs hypertrophy [54]. The collagen fibers accumulate predominantly around and between SMCs, contributing to the thickening and stiffening of the GI wall. This accumulation leads to a reduction in the gut’s resting compliance, impairing its ability to expand and contract properly [49,54]. The increased collagen deposition disrupts normal tissue architecture, making the gut less flexible and more resistant to normal physiological movements, which can exacerbate the GI complications commonly observed in diabetic conditions [10].

Building on these structural changes, our study sheds light on how pharmacological interventions may prevent or attenuate such remodeling. This is the first study to demonstrate that losartan treatment effectively prevents intestinal distension and the hypertrophy of the muscular layers in the GI wall, highlighting Ang II’s important role. In fact, Ang II, via the activation of AT_1_ receptors, plays a pivotal role in the structural alterations seen in various organs, including the cardiovascular and renal systems in diabetes [55,56]. Previous studies have shown that blocking AT_1_ receptors reduces fibrosis and muscle hypertrophy in diabetic animal models of cardiovascular disease [26]. Locally, Ang II binding to AT_1_ receptors activates multiple intracellular signaling pathways that stimulate profibrotic downstream effects, namely cellular proliferation and the accumulation of extracellular matrix [57]. This overactivation of the RAAS in diabetic patients is primarily driven by hyperglycemia, which directly and indirectly stimulates the production of Ang II [58]. The prevention of muscular hypertrophy by losartan seen herein supports the role of RAAS in these pathological changes as well as shows that blocking AT_1_ receptors may mitigate these effects in the gut, preserving normal muscle architecture and function. However, the increased mucosal thickness was not prevented by losartan. Nevertheless, a study using enalapril concluded that ACE inhibition alleviated both morphological and functional changes in the diabetic mucosa [59], showing other underlying mechanisms to be disclosed.

In contrast, the protective effects observed with losartan were not replicated with finerenone, highlighting a differential impact of targeting distinct components of the RAAS. This indicates that the observed remodeling may be more dependent on Ang II signaling than on aldosterone-mediated pathways. While MRAs have been shown to reduce fibrosis and inflammation in other tissues, such as the kidney and heart [42,44], their limited effect on the GI tract suggests a distinct regulatory mechanism and that targeting the AT1 receptor will prove a more effective strategy.

Given the known interplay between RAAS activation and oxidative stress, we next investigated how these treatments influenced redox balance in the diabetic GI tract. ROS are known to be key mediators of fibrosis induced by Ang II [60,61] and have been associated with the GI damage caused by diabetes [34]. GSH, the primary intracellular antioxidant, plays a crucial role in signaling, detoxification, and reactive species inactivation [62]. To assess the redox status in the GI tracts of diabetic animals, we measured tGSH and the GSH/GSSG ratio—key indicators of oxidative stress [63]. We also measured GSSG, which increases following oxidative stress [30]. This approach was chosen over other markers like protein carbonylation or lipid peroxidation, as those reflect mostly end-stage damage [64]. Given our short-term diabetes model, we aimed to capture early redox alterations and potential intercellular mechanisms. In these studies, diabetic animals exhibited increased local levels of tGSH and GSSG and a decreased GSH/GSSG ratio, indicating an imbalance in redox homeostasis and heightened oxidative stress in the GI tract. Only one previous study has examined local GSH levels in the GI tract in a T2D model, reporting a decrease in both tGSH and the GSH/GSSG ratio [35].

In the context of short-term STZ- induced diabetes, our study also observed elevated levels of reduced glutathione (GSH) in diabetic segments, specifically in the stomach, ileum, and colon. This increase likely represents an early adaptive response to oxidative stress, potentially mediated by the activation of the nuclear factor erythroid 2–related factor 2 (Nrf2) pathway [65]. Nrf2 is a key transcription factor that, upon activation by oxidative stress, translocates to the nucleus and upregulates the expression of various antioxidant genes, including those involved in GSH synthesis such as the glutamate-cysteine ligase catalytic subunit [66]. The activation of the Nrf2 pathway in response to oxidative stress serves as a compensatory mechanism to bolster antioxidant defenses [67]. However, in the early stages of diabetes, this response may be insufficient to counteract the excessive ROS production, leading to oxidative damage [65]. Additionally, our findings also indicate a concomitant increase in GSSG levels, leading to a decreased GSH/GSSG ratio. This suggests that the rate of GSH oxidation surpasses its synthesis or regeneration capacity, resulting in a redox imbalance favoring an oxidative environment. Such an imbalance is characteristic of diabetic conditions, in which increased ROS production overwhelms the antioxidant defense mechanisms [29]. Moreover, the hyperglycemia-induced oxidative stress in diabetes can impair the regeneration of GSH from GSSG due to the depletion of NADPH, a critical cofactor in this process. High glucose levels have been shown to inhibit glucose-6-phosphate dehydrogenase activity, reducing NADPH production and thereby hindering GSH regeneration [68].

The increased levels of GSSG in diabetes have been previously described and are related to increased levels of ROS [69,70]. The decreased GSH/GSSG ratio, which is approximately half in these diabetic samples compared to controls, aligns with findings previously reported by our group [35] and the plasmatic values described by others [70,71].

Importantly, our data reveal that pharmacological intervention with losartan significantly modulated this redox imbalance, unlike finerenone. To date, there are no other studies specifically examining the effects of losartan on the GI tract in diabetic animal models. However, research in diabetic animals has demonstrated that AT1 receptor antagonists such as losartan and valsartan, even at moderate doses, can have a profound and rapid effect in suppressing systemic ROS [72,73] and that Ang II exacerbates oxidative stress by increasing ROS production and decreasing GSH [74]. These findings highlight the potential therapeutic benefits of using these medications not only for managing blood pressure but also for mitigating oxidative damage in diabetic conditions [72,73]. Our hypothesis is that the decreased production of ROS in the gut following losartan administration does not trigger increased levels of tGSH or a reduced GSH/GSSG ratio, as observed in the STZ group. On the other hand, the lack of effect of finerenone suggests that aldosterone signaling may not be the primary driver of oxidative stress in the diabetic GI tract. Previous studies have indicated that aldosterone contributes to oxidative damage mainly through the upregulation of ROS production in cardiovascular and other tissues [25,55], but the present findings indicate that the gut may involve distinct or additional regulatory pathways, since MR blockade alone was insufficient to restore redox balance in the present study. These pathways may include hyperglycemia-induced mitochondrial dysfunction, NADPH oxidase activation [69], or alterations in local inflammatory mediators [74]. Therefore, further studies are warranted to investigate alternative sources of oxidative stress and the role of non-aldosterone-dependent mechanisms in diabetic gut pathology.

Given the central role of RAAS in both structural and oxidative alterations, we next evaluated the systemic and local activity of ACE and ACE2 to uncover potential mechanisms. We examined local ACE and ACE2 activity in the diabetic GI tract, which to the best of our knowledge had not been previously investigated. In addition to the systemic RAAS, the GI tract expresses all of the key components of the RAAS, which not only exerts a direct effect on intestinal smooth muscle function but also influences it indirectly via the myenteric plexus cholinergic neurons, affecting both local smooth muscle activity and neural communication pathways [16]. Systemically, all diabetic animals, irrespective of treatment, exhibited increased ACE and ACE2 activity, confirming previous reports that diabetes is associated with dysregulation of the RAAS [75,76]. However, the fact that the ratios of ACE activity (Z-FHL/hHL) and the ACE2/ACE ratio remained consistent across all experimental groups suggests that the N and C-domain activities are increased in proportion to each other. The same tendency is observed when examining ACE2 and ACE. These results were described previously, and the systemic upregulation of ACE2 suggests a compensatory mechanism possibly aimed at counteracting the deleterious effects of heightened ACE activity and Ang II production [77]. However, a discrepancy emerged between systemic and local RAS activity: While systemic ACE and ACE2 were both upregulated, local ACE2 activity in the GI tract remained unchanged and local ACE activity was significantly increased. This discrepancy between systemic and local RAS activity aligns with previous findings suggesting that tissue-specific RAS alterations may contribute differently to diabetic complications [55,56]. This study utilized both Z-FHL and h-HL to measure ACE activity in various tissues, highlighting how the Z-FHL/h-HL ratio can provide insights into the functional balance between the N- and C-domains [78]. Some diseases have been shown to increase or decrease this ratio [78,79]. Research on diabetic rats has shown that alterations in ACE activity, particularly in the N-domain, might contribute to tissue-specific changes in ACE activity [80]. Nevertheless, this seems to contradict our observations in the diabetic gut, where we observed that while the portions from the control group exhibited a Z-FHL/h-HL ratio notably greater than 4.5 (which is indicative of a predominant N-domain activity), there was a decrease in the Z-FHL/h-HL ratio in certain diabetic tissues (duodenum and jejunum). This reduction to values close to 2 shows a functional shift favoring enhanced C-domain activity—the domain primarily responsible for converting Ang I into Ang II. This increased local ACE activity in diabetic animals further supports the notion that Ang II is a key mediator of GI tract remodeling. Since losartan effectively prevented changes in muscular thickness and oxidative stress, it is plausible that blocking the effects of Ang II at the tissue level is essential to mitigating these alterations. From another perspective, a more balanced N-/C-domain activity might have a protective role. The N-domain breaks down Ac-SDKP [81], a peptide that helps reduce fibrosis and inflammation [82]. So, if N-domain activity decreases, Ac-SDKP may be preserved, maintaining its protective effects. This raises the possibility that, in diabetes, increased ACE activity with balanced domain interaction—rather than N-domain dominance—could help protect tissues. Still, this remains speculative.

Interestingly, while ACE activity was altered, ACE2 responses remained unchanged locally, suggesting a decoupling of the classical and alternative RAAS axes. Our finding of no significant changes in local ACE2 activity suggests that alternative pathways, independent of the ACE2/Ang-(1-7)/Mas axis, may be at play in the GI tracts of diabetic animals. Additionally, the ACE2/ACE ratio was reduced in diabetic tissues, further indicating an imbalance in the RAAS components, which favors increased Ang II production. This imbalance, not compensated by enhanced degradation, could contribute to tissue-specific pathologies. In fact, an increase in ACE activity, not accompanied by a corresponding rise in ACE2 locally, was also observed in the diabetic kidney, which was considered a key driver of renal injury [77,83].

Given that losartan’s mechanism of action focuses on preventing the binding of Ang II to its receptor, one might question the rationale for studying ACE activity in losartan-treated animals and whether any effect is to be expected. Interestingly, losartan has been shown to bind to a specific pocket on the ACE enzyme without inhibiting its catalytic activity [84]. This interaction suggested that losartan could modulate ACE function through allosteric mechanisms, potentially influencing the enzyme’s interaction with other substrates or proteins without directly affecting its enzymatic activity [84]. However, in this study, neither losartan nor finerenone altered ACE or ACE2 activity in in the serum or GI tract. Although losartan has the ability to interact with ACE, the potential influence of this interaction remains to be uncovered.

Moreover, it is important to note that this study utilized a short-term STZ-induced diabetes model. While this model is valuable for investigating early pathological changes, it may not fully capture the chronic alterations that occur in the later stages of the disease. To gain a more comprehensive understanding of the therapeutic potential of AT_1_R blockers on diabetic GI changes, we plan to extend our investigations to long-term diabetes models and evaluate the effects of ARBs after the onset of diabetes, in order to assess their curative potential. Nevertheless, the findings presented here not only advance our understanding of the local Ang II signaling involved in diabetes-related structural changes in the GI tract but also highlight the protective effects of AT_1_R blockade—offering a promising therapeutic strategy for preventing or mitigating diabetic GI remodeling.

## 4. Materials and Methods

### 4.1. Animals and Housing

This project was approved by the institutional ICBAS-UP animal welfare body (P515/2024). Thirty-two male Wistar rats, aged 10 to 12 weeks and weighing 250–350 g, were purchased from Charles River (Charles River Laboratories, Lyon, France). The animals were acclimatized and housed at the ICBAS-UP rodent facility, where they were maintained under a 12-h light/dark cycle with controlled ventilation, temperature (20–24 °C), and relative humidity (40–60%). Each pair of rats was housed in a Sealsafe Plus GR900 Tecniplast^®^ cage with appropriate bedding (Corncob Ultra 12, Ultragene, Santa Comba Dão, Portugal) and environmental enrichment, including nesting paper, paper tunnels, and a mixture of cereal seeds and flakes. All of the rats had free access to autoclaved water (two bottles *per* cage) and a laboratory rodent diet (4 RF21, Mucedola Srl, Milan, Italy). This study followed the ARRIVE guidelines (S3 Supplementary Materials—ARRIVE guidelines) for reporting experiments.

### 4.2. Diabetes Induction

1. On the day of DM induction (d0), the rats were fasted for 4 h with free access to autoclaved tap water. The STZ solution (S0130, Sigma-Aldrich, Saint Louis, Missouri, EUA) (55 mg/mL in citrate buffer pH 4.5) was prepared just prior to the injection, since a freshly prepared solution is considered to be more effective [85]. Diabetes was induced via a single intraperitoneal injection of STZ (55 mg/kg) [85], under tramadol analgesia (Tramal^®^ oral suspension, 100 mg tramadol/mL, Grünenthal; 20 mg/kg, PO), administered moments before. Rats maintained *ad libitum* access to water and food through the remaining protocol. Animals were considered diabetic if their blood glucose was ≥250 mg/dL 48 h after STZ injection. Glycemia was measured using a GlucoMen Areo GK glucometer from Menarini Diagnostics (Paço de Arcos, Portugal) (small sample size < 0.6 µL blood) and compatible test stripes. Blood glucose level in diabetic rats was measured by sampling from a tail vein at day 0 (baseline), day 2 (to confirm diabetes), day 7, and day 14. Eight diabetic rats received voluntary oral treatment with losartan (20 mg/kg) (STZ+LOS group) and another eight were voluntarily orally treated with finerenone (10 mg/kg) (STZ+FIN group), both mixed with peanut butter from the day of induction until the end of the protocol (S1 Supplementary Materials—Videos). Losartan was given at 20 mg/kg/day, a dose within the range (10–20 mg/kg) commonly used in diabetic rats [86,87]. As a previous study showed that peanut butter can reduce the systemic absorption of losartan [88], the higher dose was selected to compensate for potential reductions in bioavailability associated with the use of this vehicle. Finerenone was administered at 10 mg/kg/day, based on prior studies demonstrating that this dose produces maximal cardiorenal and vascular protective effects in diabetic rat models [89]. Importantly, the same dose has been used effectively even when co-administered with food [90]. To ensure voluntary ingestion, both losartan and finerenone were finely ground each day and dissolved in 1 mL of autoclaved drinking water. This solution was then thoroughly mixed with 3 mL of peanut butter. The final mixture was offered directly to the animals via a syringe. All of the animals, including the controls, received peanut butter to ensure consistent handling and exposure. A habituation period of 5 days was conducted prior to the induction of diabetes to train the animals to ingest the mixture voluntarily, thereby avoiding the need for oral gavage or physical restraint. Eight diabetic animals remained untreated (STZ group). Different animals of similar age and body weight (*n* = 8), that did not undergo any of these procedures, were used as the controls (CTRL group).

### 4.3. Animal Monitorization and Welfare Evaluation

The animals used in this project were monitored daily, from 11:00 AM to 1:00 PM, throughout the entire protocol, with all observations recorded in individual evaluation tables (S2 Supplementary Materials—Welfare score). Our assessment began in the maintenance room, where we evaluated coat appearance, piloerection, posture before and after a brief stimulus, signs of abdominal discomfort, and changes in breathing patterns. Next, in the observation room, with the box open inside the flow chamber, we reassessed these parameters and additionally evaluated the animals’ hydration status. The Grimace Scale was also applied as part of the evaluation in order to evaluate pain signs [91]. Monitoring continued with the weighing of each animal, which was also conducted prior to the fasting period on day 0. Food and water intake were also measured daily. To ensure hygiene and welfare, cages were changed whenever they became excessively wet due to polyuria (a classical sign of diabetes), typically daily. For animal welfare considerations, the rats were always housed in pairs per cage.

### 4.4. Tissue Harvesting

On day 14, CTRL, STZ, STZ+LOS, and STZ+FIN rats were euthanized by isoflurane overdosage followed by decapitation, using a guillotine suitable for that species (Small Guillotine, Harvard Apparatus). Whole blood was collected by the cervical wound immediately after decapitation and was left to rest at room temperature for 2 h. It was then centrifuged at 3000 rpm for 20 min, and the serum was collected and stored at −80 °C until analysis. The abdomen of each rat was opened, and the overall appearance of the viscera was evaluated, followed by the removal of the GI tract from stomach to the distal colon, sectioned just proximal to the pubic symphysis. The longitudinal length of the colon was measured, and then the GI tract was separated in four parts: cecum, colon, intestine, and stomach. The cecum was discarded and then the remaining parts were gently cleaned of their contents using Krebs–Henseleit solution (in mM: 118 NaCl; 4.8 KCl; 2.5 CaCl_2_·2H_2_O; 1.2 NaH_2_PO_4_·H_2_O; 1.2 MgSO_4_·7H_2_O; 25 NaHCO_3_; 0.02 Na_2_EDTA; 0.3 Ascorbic acid; 11 monohydrated glucose). A 1 cm portion of all parts was opened through the non-mesenteric border and laid flat to measure the circumferential perimeter. The stomach was also opened to separate the glandular and forestomach parts, with the latter being discarded.

**Figure 9:**
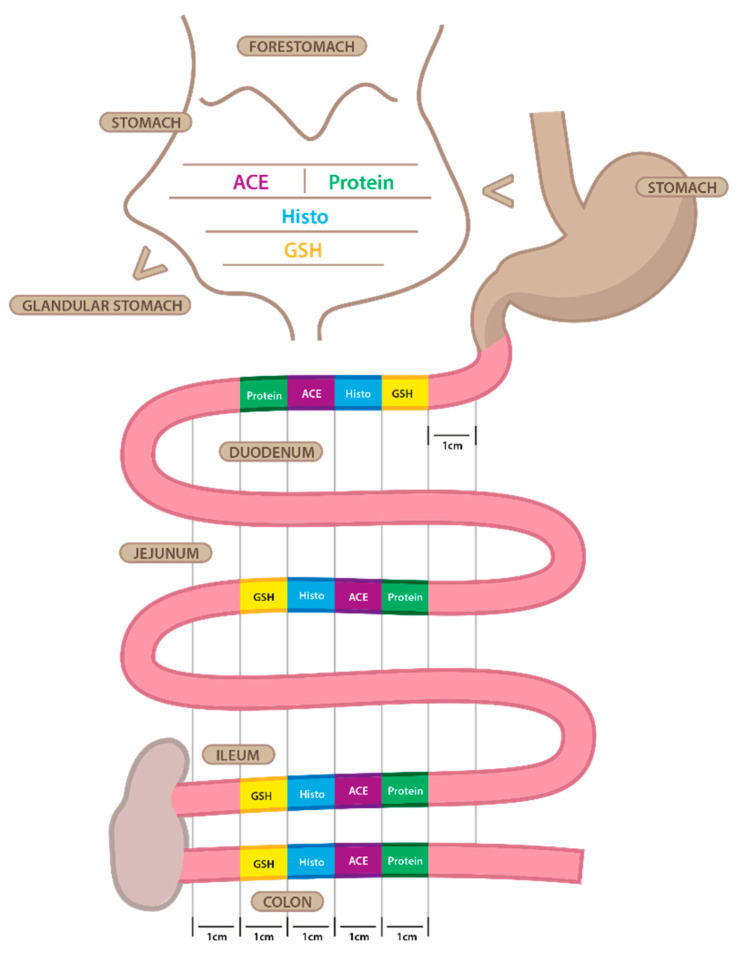
Representative illustration of the localization of the different portions of stomach, duodenum, jejunum, ileum, colon, and removed in order to perform total glutathione and oxidized glutathione quantification (GSH), histopathology (histo), angiotensin converting enzyme (ACE) and ACE2 activity (ACE) and protein quantification (protein).

A 1 cm segment from the colon, ileum, jejunum, duodenum, and stomach of the CTRL, STZ, STZ+LOS, and STZ+FIN rats was collected for ACE and ACE2 activity, histological examination, and total GSH (tGSH) and GSSG quantification (Figure 9).

### 4.5. Histology

All of the samples were dehydrated in successive ethanol solutions (70%, 96%, and 99%) and embedded in paraffin. Then, 3 µm-thick sections were cut perpendicularly to the mucosa using a microtome and mounted on sterilized glass slides. The sections were subsequently rehydrated through a graded ethanol series (99%, 96%, 70%), washed in water, and stained with hematoxylin and eosin (H&E).

**Figure 10:**
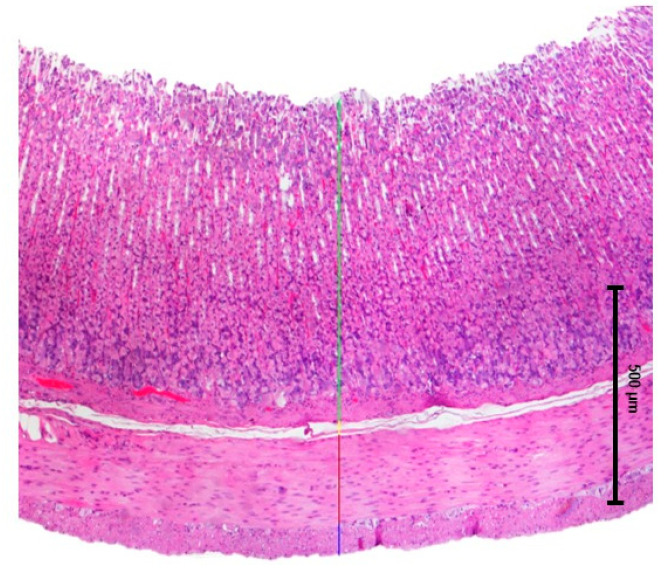
Example of how the measurements in the histopathological images were conducted: blue—longitudinal muscle, red—circular muscle, yellow—submucosa, green—mucosa.

Each section was examined under an optical microscope (Nikon Instruments, Eclipse E600, Miami, FL, USA) and photographed in representative regions at 40× (10× eyepiece × 4× objective) and 200× (10× eyepiece × 20× objective) magnification. The thickness of the mucosa, submucosa, and circular and longitudinal muscle layers was measured by the same research team member using Nikon NIS-Elements software v6.10.01 (Figure 10). For each sample, layer thickness was randomly measured at twelve different locations and averaged. Measurements were performed only on images where the entire intestinal wall was clearly visible.

### 4.6. tGSH and GSSG Quantification

For tGSH and GSSG quantification, collected samples were preserved in 1000 μL of 5% (*w*/*v*) perchloric acid. The tissues were homogenized in this solution and centrifuged at 13,000 rpm for 10 min at 4 °C. The resulting pellets were stored at −20 °C for subsequent protein quantification, while the supernatant was kept at −80 °C until analysis. tGSH and GSSG levels were determined using the DTNB-GSSG reductase recycling assay, based on a modified Ellman’s method [92]. Acidic samples were neutralized with 0.76 M potassium bicarbonate and centrifuged at 13,000 rpm for 2 min at 4 °C. The same process was applied to total GSH standards (0–15 μM). In a 96-well plate, 100 μL of each sample was mixed with 65 μL of a reagent solution containing 0.63 mM NADPH and 3.96 mM DTNB prepared in a phosphate buffer (71.5 mM Na_2_HPO_4_, 71.5 mM NaH_2_PO_4_, 0.63 mM EDTA). The mixture was incubated at 30 °C for 15 min, followed by the addition of 40 μL of glutathione reductase (10 U/mL in phosphate buffer). Absorbance was measured at 415 nm over 3 min, with readings taken every 10 s, using a Biotek PowerWave X spectrophotometer (BIOTEK, Winooski, VT, USA).

tGSH and GSSG concentrations were normalized to total protein content and expressed as nmol/mg of protein. The GSH/GSSG ratio was calculated using the following formula: GSH/GSSG = $\frac{tGSH-2\times GSSG}{GSSG}$.

The pellets obtained in the previous step were dissolved in 0.5 M NaOH, and an albumin stock solution was prepared with concentrations ranging from 0.0625 mg/mL to 1 mg/mL. After homogenization, protein levels were quantified spectrophotometrically using a Biotek PowerWave HT microplate reader (BIOTEK, Vermont, USA), following the Lowry et al. method [93], with absorbance measured at 750 nm. The Lowry method was applied to samples diluted in NaOH due to its compatibility with alkaline conditions and its sensitivity to low protein concentrations.

### 4.7. ACE and ACE2 Activity

For the enzyme activity assay, the collected tissue segments (approximately 200 mg) were homogenized in 1 mL of buffer containing 100 mM sodium borohydride (pH 7.2), 340 mM sucrose, 300 mM NaCl, and 1 mM phenylmethylsulfonyl fluoride (PMSF) inhibitor. The PMSF was initially prepared as a 200 mM concentrated solution and added to the homogenization tubes at the time of preparation to achieve a final concentration of 1 mM. Buffer preparation and sample homogenization were conducted on ice. The samples were then centrifuged at 3000 rpm for 15 min at 4 °C, and the supernatants were collected and stored at −80 °C until analysis

ACE activity was measured as previously defined [94] and Hippuryl-His-Leu (h-HL) and Z-Phe-His-Leu (Z-FHL) were used as substrates. Briefly, 10 μL of tissue homogenate was incubated at 37 °C for 10 min with 200 μL of assay solution containing either 1 mM Z-FHL or 5 mM h-HL in 100 mM potassium phosphate buffer (pH 8.3), supplemented with 300 mM NaCl and 0.1 mM ZnSO_4_. The enzymatic reaction was then stopped by adding 1.5 mL of 0.28 M NaOH. To allow the binding of o-phthaldialdehyde to the newly formed HL peptide, 100 μL of an o-phthaldialdehyde solution (20 mg/10 mL in methanol) was added, and the mixture was incubated at room temperature for 10 min, resulting in a fluorescent product. The reaction was terminated by adding 200 μL of 3 NHCl, followed by centrifugation at 3000 rpm for 5 min at 4 °C. The hydrolysis product HL was quantified fluorometrically (λ_excitation = 360 nm; λ_emission = 465 nm) using a SpectraMax Gemini EM microplate reader (Molecular Devices). ACE activity was expressed as global enzyme activity normalized to total protein concentration (nmol/min/mg of total protein) for intestinal tissue and global enzyme activity for serum (nmol/min/mL).

ACE2 activity was measured using a fluorometric kinetic assay with 20 μM Mca-APK(Dnp) as the substrate (Cat. No. BML-P163-0001, Enzo Life Sciences, Inc., Farmingdale, NY, USA). Briefly, sample homogenates (10 μL for intestinal tissue or 5 μL for serum) were preincubated at 37 °C for 5 min in a buffer containing complete Mini EDTA-free protease inhibitor (1 tablet *per* 10 mL buffer), 75 mM Tris, 1 M NaCl, 0.5 mM ZnCl_2_, and 10 μM captopril (all from Merck, Darmstadt, Germany), at pH 6.5. Incubation was performed in the presence or absence of the selective ACE2 inhibitor MLN476 (10 μM). The substrate was then added, and fluorescence was recorded every 2 min over 120 min (λ_excitation = 320 nm; λ_emission = 420 nm) using a SpectraMax Gemini EM microplate reader (Molecular Devices, San Jose, CA, USA). Fluorescence values were obtained for two hours and calculations were performed based on a fluorescence standard curve obtained with an OmniMMP^®^ fluorogenic control (Cat. No. BML-P127-0001, Enzo Life Sciences, Inc.), with the time point 0 serving as an internal blank. The maximum activity value for each sample was used and ACE2 activity was expressed as global enzyme activity normalized to total protein concentration (nmol/min/mg of total protein) for intestinal tissue, and global enzyme activity for serum (nmol/min/mL).

To evaluate the relative contribution between the activities of the N- and C- domains of ACE and between the activities of ACE2 and ACE in each sample, the following ratios were calculated and analyzed: ACE-Z-FHL/ACE-h-HL activity ratio and ACE2/ACE-Z-FHL activity ratio. It has been reported that the Z-FHL/h-HL hydrolysis rate ratio for human ACE varies depending on the domain: both domains combined exhibit an activity ratio of approximately 1, the N-domain presents a ratio of 4.5, and the C-domain presents a ratio of 0.74 [95].

Total protein quantification was performed using the Bradford method, with bovine serum albumin as the standard [96]. The Bradford method was used because samples were preserved with Triton X-100, and this method is more tolerant to the presence of detergents and provides a rapid and reliable quantification under such conditions.

### 4.8. Statistical Analysis

The GraphPad Prism^®^ 8.1.2 (Graph Pad Prism Software, Inc., San Diego, CA, USA) was used for the statistical analysis of the data. The Shapiro–Wilk test was employed to assess the normality of the data. All datasets that had *p* > 0.05 were considered to have passed the normality test. To evaluate data with normal distributions, including histological and oxidative stress, between the four experimental groups (CTRL, STZ, STZ+LOS, and STZ+FIN), an ordinary one-way ANOVA with Tukey multiple comparisons was used. Data were expressed as mean ± SD, where n refers to the number of experimental animals. To evaluate data with a non-Gaussian distribution, (ACE and ACE2 activities and respective ratios) the Kruskal–Wallis test with multiple comparisons followed by Dunn’s multiple comparisons test was used, and data were expressed and represented as a median [95% confidence limits]. In all cases, a *p* value of less than 0.05 was considered to denote a statistically significant difference.

The sample size of animals was decided using the free software Sample Size Calculator (ClinCalc.com, Chicago, IL, USA, https://clincalc.com/stats/samplesize.aspx, accessed on 12 June 2024) based on a power of 80%, a significance level of 0.05, and an estimated effect size of 4.5, derived from preliminary data on wall thickness in diabetic vs. control animals.

## 5. Conclusions

Our findings highlight the differential effects of losartan and finerenone on diabetes-induced alterations in the GI tract. The prevention of muscular hypertrophy and oxidative stress by losartan underscores the importance of AT_1_ receptor-mediated mechanisms in these pathological changes. In contrast, the lack of effect of finerenone suggests that MR blockade alone is insufficient to counteract the remodeling and oxidative imbalances observed. Additionally, the observed discrepancies between systemic and local RAAS activity further emphasize the complexity of RAAS regulation in diabetes.

These findings suggest that angiotensin receptor blockers like losartan may help prevent diabetes-induced remodeling of the GI tract, whereas mineralocorticoid receptor antagonists do not provide the same level of protection. Future studies should further explore the precise mechanisms underlying tissue-specific RAAS regulation and assess the potential of angiotensin receptor blockers not only as a preventive strategy, as observed in this study, but also as possible therapeutic intervention.

## 6. Patents

M. Duarte-Araújo, M. Esteves-Monteiro, and M. Morato. “Repurposing the use of angiotensin converting enzyme inhibitors and angiotensin II receptor antagonists to prevent and/or treat gastrointestinal complications associated with Diabetes mellitus”, International Patent Application No. PCT/IB2025/052714, filed by Universidade do Porto and REQUIMTE—Rede de Química e Tecnologia, priority filing PT 119319 (Portugal), 14 March 2024.

## Data Availability

Dataset available on request from the authors.

## Supplementary Materials

The supporting information can be downloaded at: https://www.mdpi.com/article/10.3390/ijms26136294/s1.

## References

[1] World Health Organization (2016). Global Report on Diabetes.

[2] DiMeglio L.A., Evans-Molina C., Oram R.A. (2018). Type 1 Diabetes. Lancet.

[3] Chatterjee S., Khunti K., Davies M.J. (2017). Type 2 Diabetes. Lancet.

[4] Rees D.A., Alcolado J.C. (2005). Animal Models of Diabetes Mellitus. Diabet. Med..

[5] Furman B.L. (2021). Streptozotocin-Induced Diabetic Models in Mice and Rats. Curr. Protoc..

[6] Du Y.T., Rayner C.K., Jones K.L., Talley N.J., Horowitz M. (2018). Gastrointestinal Symptoms in Diabetes: Prevalence, Assessment, Pathogenesis, and Management. Diabetes Care.

[7] Chandrasekharan B., Srinivasan S. (2007). Diabetes and the Enteric Nervous System. Neurogastroenterol. Motil..

[8] Fan X., Wang Y., Cao J., Yu J., Tian J., Mi J. (2025). Clinical study status of diabetic gastrointestinal diseases. Front. Endocrinol..

[9] Krishnan B., Babu S., Walker J., Walker A.B., Pappachan J.M. (2013). Gastrointestinal Complications of Diabetes Mellitus. World J. Diabetes.

[10] Zhao M., Liao D., Zhao J. (2017). Diabetes-Induced Mechanophysiological Changes in the Small Intestine and Colon. World J. Diabetes.

[11] Boland B.S., Edelman S.V., Wolosin J.D. (2013). Gastrointestinal Complications of Diabetes. Endocrinol. Metab. Clin. N. Am..

[12] Goyal R.K., Spiro H.M. (1971). Gastrointestinal Manifestations of Diabetes Mellitus. Med. Clin. N. Am..

[13] Bulc M., Całka J., Palus K. (2020). Effect of Streptozotocin-Inducted Diabetes on the Pathophysiology of Enteric Neurons in the Small Intestine Based on the Porcine Diabetes Model. Int. J. Mol. Sci..

[14] Dedeli O., Pakyuz S.C., Daban U.K., Kipcak S., Sari D. (2015). Prevalence of Gastrointestinal Symptoms and Its Effect on Quality of Life among Patients with Diabetes Mellitus. Am. J. Nurs. Res..

[15] Garg M., Angus P.W., Burrell L.M., Herath C., Gibson P.R., Lubel J.S. (2012). Review Article: The Pathophysiological Roles of the Renin—Angiotensin System in the Gastrointestinal Tract. Aliment. Pharmacol. Ther..

[16] Zizzo M.G., Serio R. (2023). The Renin–Angiotensin System in Gastrointestinal Functions. Angiotensin.

[17] Atlas S.A. (2007). The Renin-Angiotensin Aldosterone System: Pathophysiological Role and Pharmacologic Inhibition. J. Manag. Care Pharm..

[18] Martyniak A., Tomasik P.J. (2022). A New Perspective on the Renin-Angiotensin System. Diagnostics.

[19] Guang C., Phillips R.D., Jiang B., Milani F. (2012). Three Key Proteases—Angiotensin-I-Converting Enzyme (ACE), ACE2 and Renin—Within and beyond the Renin-Angiotensin System. Arch. Cardiovasc. Dis..

[20] Lubbe L., Cozier G.E., Oosthuizen D., Acharya K.R., Sturrock E.D. (2020). ACE2 and ACE: Structure-Based Insights into Mechanism, Regulation and Receptor Recognition by SARS-CoV. Clin. Sci..

[21] Burrell L.M., Johnston C.I., Tikellis C., Cooper M.E. (2004). ACE2, a New Regulator of the Renin–Angiotensin System. Trends Endocrinol. Metab..

[22] Bernardi S., Michelli A., Zuolo G., Candido R., Fabris B. (2016). Update on RAAS Modulation for the Treatment of Diabetic Cardiovascular Disease. J. Diabetes Res..

[23] Saravi B., Li Z., Lang C.N., Schmid B., Lang F.K., Grad S., Alini M., Richards R.G., Schmal H., Südkamp N. (2021). The Tissue Renin-Angiotensin System and Its Role in the Pathogenesis of Major Human Diseases: Quo Vadis?. Cells.

[24] Esteves-Monteiro M., Menezes-Pinto D., Ferreira-Duarte M., Dias-Pereira P., Morato M., Duarte-Araújo M. (2022). Histomorphometry Changes and Decreased Reactivity to Angiotensin II in the Ileum and Colon of Streptozotocin-Induced Diabetic Rats. Int. J. Mol. Sci..

[25] Rahimi Z., Moradi M., Nasri H. (2014). A Systematic Review of the Role of Renin Angiotensin Aldosterone System Genes in Diabetes Mellitus, Diabetic Retinopathy and Diabetic Neuropathy. J. Res. Med. Sci..

[26] Batista J.P.T., de Faria A.O.V., Ribeiro T.F.S., Simões e Silva A.C. (2023). The Role of Renin–Angiotensin System in Diabetic Cardiomyopathy: A Narrative Review. Life.

[27] Ustundag B., Canatan H., Cinkilince N., Hali H. (2000). Angiotensin Converting Enzyme (ACE) Activity Levels in Insulin-Independent Diabetes Mellitus and Effect of ACE Levels on Diabetic Patients with Nephropathy. Cell Biochem. Funct..

[28] Singh V.P., Bali A., Singh N., Jaggi A.S. (2014). Advanced Glycation End Products and Diabetic Complications. Korean J. Physiol. Pharmacol..

[29] Giacco F., Brownlee M. (2010). Oxidative Stress and Diabetic Complications. Circ. Res..

[30] Wu G., Lupton J.R., Turner N.D., Fang Y.-Z., Yang S. (2004). Glutathione Metabolism and Its Implications for Health. J. Nutr..

[31] Townsend D.M., Tew K.D., Tapiero H. (2003). The Importance of Glutathione in Human Disease. Biomed. Pharmacother..

[32] Zitka O., Skalickova S., Gumulec J., Masarik M., Adam V., Hubalek J., Trnkova L., Kruseova J., Eckschlager T., Kizek R. (2012). Redox Status Expressed as GSH:GSSG Ratio as a Marker for Oxidative Stress in Paediatric Tumour Patients. Oncol. Lett..

[33] Kuyvenhoven J.P., Meinders A.E. (1999). Oxidative Stress and Diabetes Mellitus Pathogenesis of Long-Term Complications. Eur. J. Intern. Med..

[34] Kashyap P., Farrugia G. (2011). Oxidative Stress: Key Player in Gastrointestinal Complications of Diabetes. Neurogastroenterol. Motil..

[35] Esteves-Monteiro M., Ferreira-Duarte M., Vitorino-Oliveira C., Costa-Pires J., Oliveira S., Matafome P., Morato M., Dias-Pereira P., Costa V.M., Duarte-Araújo M. (2024). Oxidative Stress and Histomorphometric Remodeling: Two Key Intestinal Features of Type 2 Diabetes in Goto–Kakizaki Rats. Int. J. Mol. Sci..

[36] Guthrie R.A., Guthrie D.W. (2004). Pathophysiology of Diabetes Mellitus. Crit. Care Nurs. Q..

[37] Ghasemi A., Jeddi S. (2023). Streptozotocin as a Tool for Induction of Rat Models of Diabetes: A Practical Guide. EXCLI J..

[38] Davis F.B., Davis P.J. (1991). Water Metabolism in Diabetes Mellitus. Am. J. Med..

[39] Havel P.J., Sindelar D.K., Baskin D.G., Dallman M.F. (2000). Effects of Streptozotocin-Induced Diabetes and Insulin Treatment on the Hypothalamic Melanocortin System and Muscle Uncoupling Protein 3 Expression in Rats. Diabetes.

[40] Chen P., Zhao J., Gregersen H. (2012). Up-Regulated Expression of Advanced Glycation End-Products and Their Receptor in the Small Intestine and Colon of Diabetic Rats. Dig. Dis. Sci..

[41] Weil E.J., Fufaa G., Jones L.I., Lovato T., Lemley K.V., Hanson R.L., Knowler W.C., Bennett P.H., Yee B., Myers B.D. (2013). Effect of Losartan on Prevention and Progression of Early Diabetic Nephropathy in American Indians With Type 2 Diabetes. Diabetes.

[42] Zhai S., Ma B., Chen W., Zhao Q. (2024). A Comprehensive Review of Finerenone—A Third-Generation Non-Steroidal Mineralocorticoid Receptor Antagonist. Front. Cardiovasc. Med..

[43] Laakso M., Karjalainen L., Lempiäinen-Kuosa P. (1996). Effects of Losartan on Insulin Sensitivity in Hypertensive Subjects. Hypertension.

[44] Veneti S., Tziomalos K. (2021). The Role of Finerenone in the Management of Diabetic Nephropathy. Diabetes Ther..

[45] Forrest A., Huizinga J.D., Wang X.Y., Liu L.W., Parsons M. (2007). Increase in Stretch-Induced Rhythmic Motor Activity in the Diabetic Rat Colon Is Associated with Loss of ICC of the Submuscular Plexus. Am. J. Physiol. Gastrointest. Liver Physiol..

[46] Jervis L., Levim R. (1996). Anatomic Adaptation of the Alimentary Tract of the Rat to the Hyperphagia of Chronic Alloxan-Diabetes. Nature.

[47] Cripps A.W., Williams V.J. (2008). The Effect of Pregnancy and Lactation on Food Intake, Gastrointestinal Anatomy and the Absorptive Capacity of the Small Intestine in the Albino Rat.

[48] Brobeck J.R., Tepperman J., Long C.N.H. (1943). Experimental Hypothalamic Hyperphagia in the Albino Rat. Yale J. Biol. Med..

[49] Zhao J., Yang J., Gregersen H. (2003). Biomechanical and Morphometric Intestinal Remodelling during Experimental Diabetes in Rats. Diabetologia.

[50] Fischer K.D., Dhanvantari S., Drucker D.J., Brubaker P.L., Kirk D., Dhanvantari S., Daniel J., Brubaker P.L. (1997). Intestinal Growth Is Associated with Elevated Levels of Glucagon-like Peptide 2 in Diabetic Rats. Am. J. Physiol..

[51] Noda T., Iwakiri R., Fujimoto K., Yoshida T., Utsumi H., Sakata H., Hisatomi A., Aw T.Y. (2001). Suppression of Apoptosis Is Responsible for Increased Thickness of Intestinal Mucosa in Streptozotocin-Induced Diabetic Rats. Metabolism.

[52] Sha H., Tong X., Zhao J. (2018). Abnormal Expressions of AGEs, TGF- β1, BDNF and Their Receptors in Diabetic Rat Colon—Associations with Colonic Morphometric and Biomechanical Remodeling. Sci. Rep..

[53] Zhao J., Chen P., Gregersen H. (2013). Morpho-Mechanical Intestinal Remodeling in Type 2 Diabetic GK Rats-Is It Related to Advanced Glycation End Product Formation?. J. Biomech..

[54] Siegman M.J., Eto M., Butler T.M. (2016). Remodeling of the Rat Distal Colon in Diabetes: Function and Ultrastructure. Am. J. Physiol. Cell Physiol..

[55] Lim H.S., MacFadyen R.J., Lip G.Y.H. (2004). Diabetes Mellitus, the Renin-Angiotensin-Aldosterone System, and the Heart. Arch. Intern. Med..

[56] Rein J., Bader M. (2017). Renin-Angiotensin System in Diabetes. Protein Pept. Lett..

[57] Murphy A.M., Wong A.L., Bezuhly M. (2015). Modulation of Angiotensin II Signaling in the Prevention of Fibrosis. Fibrogenesis Tissue Repair..

[58] Hsueh W.A., Wyne K. (2011). Renin-Angiotensin-Aldosterone System in Diabetes and Hypertension. J. Clin. Hypertens..

[59] Jaworska K., Kopacz W., Koper M., Szudzik M., Gawryś-Kopczyńska M., Konop M., Hutsch T., Chabowski D., Ufnal M. (2022). Enalapril Diminishes the Diabetes-Induced Changes in Intestinal Morphology, Intestinal RAS and Blood SCFA Concentration in Rats. Int. J. Mol. Sci..

[60] Ruiz-ortega M., Carvajal G. (2004). Angiotensin II: A Key Factor in the Inflammatory and Fibrotic Response in Kidney Diseases. Nephrol. Dial. Transplant..

[61] Macconi D., Remuzzi G., Benigni A. (2014). Key Fibrogenic Mediators: Old Players. Renin–Angiotensin System. Kidney Int. Suppl..

[62] Aquilano K., Baldelli S., Ciriolo M.R. (2014). Glutathione: New Roles in Redox Signaling for an Old Antioxidant. Front. Pharmacol..

[63] Forman H.J., Zhang H., Rinna A. (2009). Glutathione: Overview of Its Protective Roles, Measurement, and Biosynthesis. Mol. Aspects Med..

[64] Baynes J.W., Thorpe S.R. (1999). Role of Oxidative Stress in Diabetic Complications: A New Perspective on an Old Paradigm. Diabetes.

[65] Ebrahimi R., Mohammadpour A., Medoro A., Davinelli S., Saso L., Miroliaei M. (2025). Exploring the Links between Polyphenols, Nrf2, and Diabetes: A Review. Biomed. Pharmacother..

[66] Ma Q. (2013). Role of Nrf2 in Oxidative Stress and Toxicity. Annu. Rev. Pharmacol. Toxicol..

[67] David J.A., Rifkin W.J., Rabbani P.S., Ceradini D.J. (2017). The Nrf2/Keap1/ARE Pathway and Oxidative Stress as a Therapeutic Target in Type II Diabetes Mellitus. J. Diabetes Res..

[68] Díaz-Flores M., Ibáñez-Hernández M.A., Galván R.E., Gutiérrez M., Durán-Reyes G., Medina-Navarro R., Pascoe-Lira D., Ortega-Camarillo C., Vilar-Rojas C., Cruz M. (2006). Glucose-6-Phosphate Dehydrogenase Activity and NADPH/NADP+ Ratio in Liver and Pancreas Are Dependent on the Severity of Hyperglycemia in Rat. Life Sci..

[69] Lutchmansingh F.K., Hsu J.W., Bennett F.I., Badaloo A.V., McFarlane-Anderson N., Gordon-Strachan G.M., Wright-Pascoe R.A., Jahoor F., Boyne M.S. (2018). Glutathione Metabolism in Type 2 Diabetes and Its Relationship with Microvascular Complications and Glycemia. PLoS ONE.

[70] De Mattia G., Bravi M.C., Laurenti O., Cassone-Faldetta M., Armiento A., Ferri C., Balsano F. (1998). Influence of Reduced Glutathione Infusion on Glucose Metabolism in Patients with Non—Insulin-Dependent Diabetes Mellitus. Metabolism.

[71] Calabrese V., Cornelius C., Leso V., Trovato-Salinaro A., Ventimiglia B., Cavallaro M., Scuto M., Rizza S., Zanoli L., Neri S. (2012). Oxidative Stress, Glutathione Status, Sirtuin and Cellular Stress Response in Type 2 Diabetes. Biochim. Biophys. Acta (BBA)-Mol. Basis Dis..

[72] Lodovici M., Bigagli E., Tarantini F., Di Serio C., Raimondi L. (2015). Losartan Reduces Oxidative Damage to Renal DNA and Conserves Plasma Antioxidant Capacity in Diabetic Rats. Exp. Biol. Med..

[73] Dandona P., Kumar V., Aljada A., Ghanim H., Syed T., Hofmayer D., Mohanty P., Tripathy D., Garg R. (2003). Angiotensin II Receptor Blocker Valsartan Suppresses Reactive Oxygen Species Generation in Leukocytes, Nuclear Factor-ΚB, in Mononuclear Cells of Normal Subjects: Evidence of an Antiinflammatory Action. J. Clin. Endocrinol. Metab..

[74] Yang N., Gonzalez-Vicente A., Garvin J.L. (2020). Angiotensin II-Induced Superoxide and Decreased Glutathione in Proximal Tubules: Effect of Dietary Fructose. Am. J. Physiol.-Ren. Physiol..

[75] Batlle D., Jose Soler M., Ye M. (2010). ACE2 and Diabetes: ACE of ACEs?. Diabetes.

[76] Feman S., Mericle R.A., Reed G.W., May J.M., Workman R.J. (1993). Serum angiotensin converting enzyme in diabetic patients. Am. J. Med. Sci..

[77] Yamaleyeva L.M., Gilliam-Davis S., Almeida I., Brosnihan K.B., Lindsey S.H., Chappell M.C. (2012). Differential Regulation of Circulating and Renal ACE2 and ACE in Hypertensive MRen2.Lewis Rats with Early-Onset Diabetes. Am. J. Physiol.-Ren. Physiol..

[78] Enyedi E.E., Petukhov P.A., Kozuch A.J., Dudek S.M., Toth A., Fagyas M., Danilov S.M. (2024). ACE Phenotyping in Human Blood and Tissues: Revelation of ACE Outliers and Sex Differences in ACE Sialylation. Biomedicines.

[79] Danilov S.M., Tikhomirova V.E., Metzger R., Naperova I.A., Bukina T.M., Goker-Alpan O., Tayebi N., Gayfullin N.M., Schwartz D.E., Samokhodskaya L.M. (2018). ACE Phenotyping in Gaucher Disease. Mol. Genet. Metab..

[80] Ronchi F.A., Irigoyen M.-C., Casarini D.E. (2007). Association of Somatic and N-Domain Angiotensin-Converting Enzymes from Wistar Rat Tissue with Renal Dysfunction in Diabetes Mellitus. J. Renin-Angiotensin-Aldosterone Syst..

[81] Anthony C.S., Corradi H.R., Schwager S.L.U., Redelinghuys P., Georgiadis D., Dive V., Acharya K.R., Sturrock E.D. (2010). The N Domain of Human Angiotensin-I-Converting Enzyme. J. Biol. Chem..

[82] Sharma R.K., Douglas R.G., Louw S., Chibale K., Sturrock E.D. (2012). New Ketomethylene Inhibitor Analogues: Synthesis and Assessment of Structural Determinants for N-Domain Selective Inhibition of Angiotensin-Converting Enzyme. Biol. Chem..

[83] Mizuiri S., Hemmi H., Arita M., Ohashi Y., Tanaka Y., Miyagi M., Sakai K., Ishikawa Y., Shibuya K., Hase H. (2008). Expression of ACE and ACE2 in Individuals With Diabetic Kidney Disease and Healthy Controls. Am. J. Kidney Dis..

[84] Bezerra E.M., de Alvarenga É.C., dos Santos R.P., de Sousa J.S., Fulco U.L., Freire V.N., Albuquerque E.L., da Costa R.F. (2022). Losartan as an ACE Inhibitor: A Description of the Mechanism of Action through Quantum Biochemistry. RSC Adv..

[85] Eleazu C.O., Eleazu K.C., Chukwuma S., Essien U.N. (2013). Review of the Mechanism of Cell Death Resulting from Streptozotocin Challenge in Experimental Animals, Its Practical Use and Potential Risk to Humans. J. Diabetes Metab. Disord..

[86] Cavusoglu T., Karadeniz T., Cagiltay E., Karadeniz M., Yigitturk G., Acikgoz E., Uyanikgil Y., Ates U., Tuglu M., Erbas O. (2015). The Protective Effect of Losartan on Diabetic Neuropathy in a Diabetic Rat Model. Exp. Clin. Endocrinol. Diabetes.

[87] Kamper M., Tsimpoukidi O., Chatzigeorgiou A., Lymberi M., Kamper E.F. (2010). The Antioxidant Effect of Angiotensin II Receptor Blocker, Losartan, in Streptozotocin-Induced Diabetic Rats. Transl. Res..

[88] Diogo L.N., Faustino I.V., Afonso R.A., Pereira S.A., Monteiro E.C., Santos A.I. (2015). Voluntary Oral Administration of Losartan in Rats. J. Am. Assoc. Lab. Anim. Sci..

[89] Sanz-Gómez M., Manzano-Lista F.J., Vega-Martín E., González-Moreno D., Alcalá M., Gil-Ortega M., Somoza B., Pizzamiglio C., Ruilope L.M., Aránguez I. (2023). Finerenone Protects against Progression of Kidney and Cardiovascular Damage in a Model of Type 1 Diabetes through Modulation of Proinflammatory and Osteogenic Factors. Biomed. Pharmacother..

[90] Lima Posada I., Soulié M., Stephan Y., Palacios Ramirez R., Bonnard B., Nicol L., Pitt B., Kolkhof P., Mulder P., Jaisser F. (2024). Nonsteroidal Mineralocorticoid Receptor Antagonist Finerenone Improves Diastolic Dysfunction in Preclinical Nondiabetic Chronic Kidney Disease. J. Am. Heart Assoc..

[91] Leung V., Zhang E., Pang D.S. (2016). Real-Time Application of the Rat Grimace Scale as a Welfare Refinement in Laboratory Rats. Sci. Rep..

[92] Bastos M.L., Carvalho M., Milhazes N., Remiro F., Borges F., Fernandes E., Amado F., Monks T.J., Carvalho F. (2004). Hepatotoxicity of 3, 4-Methylenedioxyamphetamine and Methyldopamine in Isolated Rat Hepatocytes: Formation of Glutathione Conjugates. Arch. Toxicol..

[93] Lowry O.H., Rosebrough N.J., Farr A.L., Randall R.J. (1951). Protein Measurement with the Folin Phenol Reagent. J. Biol. Chem..

[94] Ferreira-Duarte M., Oliveira L.C.G., Quintas C., Esteves-Monteiro M., Duarte-Araújo M., Sousa T., Casarini D.E., Morato M. (2023). ACE and ACE2 Catalytic Activity in the Fecal Content along the Gut. Neurogastroenterol. Motil..

[95] Williams T.A., Danilov S., Alhenc-Gelas F., Soubrier F. (1996). A Study of Chimeras Constructed with the Two Domains of Angiotensin I-Converting Enzyme. Biochem. Pharmacol..

[96] Bradford M.M. (1976). A Rapid and Sensitive Method for the Quantitation of Microgram Quantities of Protein Utilizing the Principle of Protein-Dye Binding. Anal. Biochem..

