# Supplementary material for: Differential Effects of Losartan and Finerenone on Diabetic Remodeling, Oxidative Stress and ACE Activity in the Gastrointestinal Tract of Streptozotocin-Induced Diabetic Rats
Source: Int J Mol Sci. 2025 Jun 29;26(13):6294. doi: 10.3390/ijms26136294 (PMC12249876; doi:10.3390/ijms26136294)
Supplement: Supplementary file 1 [file ijms-26-06294-s001.zip › S1_Supplementary Material_Videos.pdf]

S1

Video of voluntary oral administration of losartan

S2

Video of voluntary oral administration of finerenone
