# Supplementary material for: Differential Effects of Losartan and Finerenone on Diabetic Remodeling, Oxidative Stress and ACE Activity in the Gastrointestinal Tract of Streptozotocin-Induced Diabetic Rats
Source: Int J Mol Sci. 2025 Jun 29;26(13):6294. doi: 10.3390/ijms26136294 (PMC12249876; doi:10.3390/ijms26136294)
Supplement: Supplementary file 1 [file ijms-26-06294-s001.zip › S2_Supplementary Material_Welfare score.pdf]

DiaPETis

DiaPETis	
ANIMAL:	
GROUP:	

Fasting	
Date:	
Hour:	

INDUCTION	
Date:	
Drug	

				Protocol days																
				D -1	D 0	D 1	D 2	D 3	D 4	D 5	D 6	D 7	D 8	D 9	D 10	D 11	D 12	D 13	D 14	D 15
1. Physiological state / Body functions	Body weight (grams)																			
	Food intake (grams)																			
	Water intake (mL)																			
	Fecal pellets count																			
	Glycemia																			
	Respiration	Normal	0																	
		Slightly laboured breathing	1																	
		Very laboured breathing	2																	
2. Appearance / Physical state	Body weight	Normal	0																	
		5-10%	1																	
		>10%	2																	
	Grooming	Normal	0																	
		Lack of grooming	1																	
	Eyes	normal	0																	
		Semi-closed	1																	
		Closed*	2																	
	Dehydration("Pinched" features) - skin tenting	normal skin tent	0																	
		Skin tent present on dorsum	1																	
		Failure to right itself	2																	
	Hunching	Normal posture	0																	
		Hunched posture	1																	
	Piloerection	None	0																	
		Presence of piloerection	1																	
3. Environment	Cage	Normal faeces to slightly soft	0																	
		Diarrhoea	1																	
		Normal	0																	
		Wet bedding **	1																	
		Use of nesting material	0																	
		Untouched nesting material	1																	
4. Behavior	Mobility	Normal	0																	
		Reluctance to move	1																	
		Lethargy /apathy	2																	
		Persistent Imobility <24h	3																	
		Imobility >24h	HEP																	
	Vocalization	None	0																	
		Provoked vocalization (on handling)	1																	
		Unprovoked vocalization (spontane	2																	
	Social interaction	Normal	0																	
		Isolated from social group	1																	
		Agressive attitude	2																	
	Stress indicators	Barbering	1																	
		Stereotypic behaviour**	2																	
5. Procedure-specific indicator	Loss of Body weight	0-3%	0																	
		4-10%	1																	
		11-20%	2																	
		20-25 %	3																	
		No BW recovery	HEP																	
	Stool consistency	Normal	0																	
		Soft /diarrhea	1																	
		diarrhea with blood	2																	
	Gastrointestinal feature	Normal	0																	
		Abdominal constrictions	1																	
		Distended abdomen/ swollen	2																	
			Total																	

OTHER OBSERVATIONS:

OPERATOR SIGNATURE

* Anhalgesia required
** Consult Veterinary staff
HEP, humane endpoint

SCORE	
0 a 5	Normal or slightly changed
5 a 15	Increase vigilance
15 a 25	Increase vigilance and correct altered parameters (↑ analgesia, reh
> 25	Consider Endpoints
Any parameter =	Immediate evaluation. Consider Endpoints
